# Synthesis and fungicidal activity of pyrazole derivatives containing 1,2,3,4-tetrahydroquinoline

**DOI:** 10.1186/s13065-016-0186-8

**Published:** 2016-07-04

**Authors:** Peng Lei, Xuebo Zhang, Yan Xu, Gaofei Xu, Xili Liu, Xinling Yang, Xiaohe Zhang, Yun Ling

**Affiliations:** Department of Applied Chemistry, College of Science, China Agricultural University, Beijing, 100193 China; Department of Plant Pathology, China Agricultural University, Beijing, 100193 China

**Keywords:** Pyrazole, 1,2,3,4-tetrahydroquinoline, Synthesis, Fungicidal activity, Wheat take-all

## Abstract

**Background:**

Take-all of wheat, caused by the soil-borne fungus *Gaeumannomyces graminis* var. *tritici*, is one of the most important and widespread root diseases. Given that take-all is still hard to control, it is necessary to develop new effective agrochemicals. Pyrazole derivatives have been often reported for their favorable bioactivities. In order to discover compounds with high fungicidal activity and simple structures, 1,2,3,4-tetrahydroquinoline, a biologically active group of natural products, was introduced to pyrazole structure. A series of pyrazole derivatives containing 1,2,3,4-tetrahydroquinoline were synthesized, and their fungicidal activities were evaluated.

**Results:**

The bioassay results demonstrated that the title compounds displayed obvious fungicidal activities at a concentration of 50 μg/mL, especially against *V. mali*, *S. sclerotiorum* and *G. graminis* var. *tritici*. The inhibition rates of compounds **10d**, **10e**, **10h**, **10i** and **10j** against *G. graminis* var. *tritici* were all above 90 %. Even at a lower concentration of 16.7 μg/mL, compounds **10d** and **10e** exhibited satisfied activities of 100 % and 94.0 %, respectively. It is comparable to that of the positive control pyraclostrobin with 100 % inhibition rate.

**Conclusion:**

A series of pyrazole derivatives containing 1,2,3,4-tetrahydroquinoline were synthesized and their structures were confirmed by ^1^H NMR, ^13^C NMR, IR spectrum and HRMS or elemental analysis. The crystal structure of compound **10g** was confirmed by X-ray diffraction. Bioassay results indicated that all title compounds exhibited obvious fungicidal activities. In particular, compounds **10d** and **10e** showed comparable activities against *G. graminis* var. *tritici* with the commercial fungicide pyraclostrobin at the concentration of 16.7 μg/mL.

**Graphical abstract:**
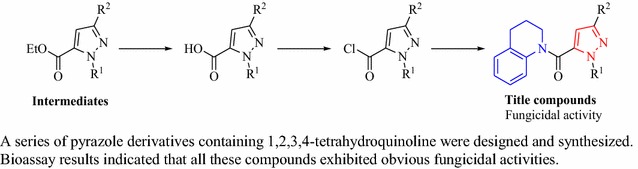
A series of pyrazole derivatives containing 1,2,3,4-tetrahydroquinoline were designed and synthesized. Bioassay results indicated that all these compounds exhibited obvious fungicidal activities.

**Electronic supplementary material:**

The online version of this article (doi:10.1186/s13065-016-0186-8) contains supplementary material, which is available to authorized users.

## Background

Wheat (*Triticum aestivum*) is one of the most important crops in the world. Take-all of wheat, caused by the soil-borne fungus *Gaeumannomyces graminis* var. *tritici*, is one of the most serious and widespread root diseases [1, 2]. The pathogen infects the roots of susceptible plants, resulting in black necrotic, plant stunting, white heads, and etc. [3, 4]. It reduces the grain yield from 20 % up to 50 %. Unfortunately, the control of take-all is still a huge problem. And the application of agrochemicals is currently the most effective method [5]. However, existing chemical control agents, such as silthiopham, were not financially affordable for the control of wheat take-all [6]. Hence, it is necessary to develop effective and inexpensive agents to replace the conventional agrochemicals.

**Fig. 1:**
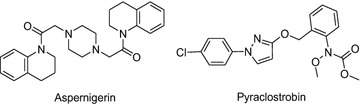
The structures of aspernigerin and pyraclostrobin

Introducing active groups of natural products is an effective and important method for the discovery of new agrochemicals [7, 8]. 1,2,3,4-tetrahydroquinoline (THQ), widely existing in natural products [9, 10], has been often reported for its favorable bioactivities, such as anticancer [11, 12], antibacterial [13, 14], antifungal [15, 16] activities, and so on. For example, aspernigerin (Fig. 1), isolated from the extract of a culture of *Aspergillus niger* IFB-E003, exhibited favorable cytotoxic to the tumor cell lines [17], and certain fungicidal activities, insecticidal activities and herbicidal activities [18, 19].

In recent years, pyrazole derivatives have attracted tremendous attention owing to their excellent bioactivities [20–22]. Pyraclostrobin (Fig. 1) discovered by BASF is a commercial fungicide containing pyrazole structure. It came to the market in 2002. Given its wide fungicidal spectrum, pyraclostrobin had achieved a total sale of $800 million in 2012, ranked the second in the world. [23]. Besides, pyrazole derivatives were also reported to possess insecticidal activities [24, 25], herbicidal activities [26], and anticancer activities [27, 28].

It is an effective method to develop new green agrochemicals by introducing active groups of natural products to known active sub-structures. As above mentioned, THQ is an important active group of natural products. In order to find highly biologically active lead compounds with simple structures, THQ was introduced to the known active sub-substructure of pyrazole compounds using intermediate derivatization methods (IDM) [29]. A series of pyrazole derivatives containing 1,2,3,4-tetrahydroquinoline were synthesized, and their activities were evaluated in this study. Biological assays revealed that some compounds exhibited good fungicidal activities. Especially, they displayed excellent activities against *G. graminis* var. *tritici*.

## Results and discussion

### Synthesis

**Scheme 1:**
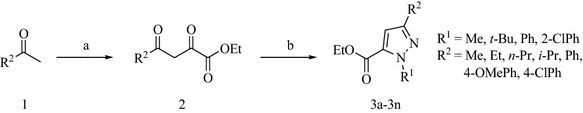
Synthetic route of intermediates **3a–3n**. Reagents and conditions: (a) CH_3_CH_2_ONa, CH_3_CH_2_OH, diethyl oxalate, room temperature (r.t.), 2 h; (b) glacial acetic acid, r.t., 0.5 h; substituted hydrazine, r.t., overnight

The synthetic procedure of intermediates **3a–3n** is shown in Scheme 1 [30]. By using Claisen condensation in the presence of sodium ethoxide, substituted ketone **1** reacted with diethyl oxalate to afford the β-ketoester intermediate **2**. With glacial acetic acid acidification, compound **2** was reacted with substituted hydrazine via Knorr reaction to obtain the intermediates **3a–3n**. This method has two advantages. Firstly, ethyl 5-pyrazolecarboxylate compounds were synthesized simply through a “one-pot” process. Secondly, the reaction proceeds well at ambient temperature.

**Scheme 2:**
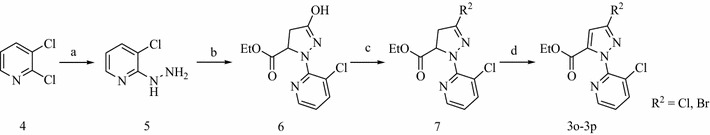
Synthetic route of intermediates **3o–3p**. Reagents and conditions: (a) NH_2_NH_2_·H_2_O (80 %), reflux, 5 h; (b) CH_3_CH_2_ONa, CH_3_CH_2_OH, reflux, 10 min, then diethyl maleate, reflux, 30 min; (c) POCl_3_ or POBr_3_, CH_3_CN, reflux, 5 h; (d) H_2_SO_4_, CH_3_CN, r.t., 10 min, then K_2_S_2_O_8_, reflux, 4 h

Synthesis of compounds **3o–3p** is carried out following a different method [31, 32] and the procedure was shown in Scheme 2. 2,3-dichloropyridine **4** reacted with hydrazine hydrate (80 %) to yield the intermediate **5**, which underwent cyclization with diethyl maleate to give the intermediate **6**. The reaction of **6** with phosphorus oxychloride or phosphorus oxybromide afforded the chlorine or bromine substituted compound **7,** which was then oxidized to give the intermediates **3o–3p**.

**Scheme 3:**
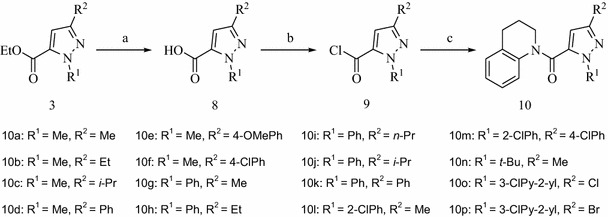
Synthetic route of the target compounds **10**. Reagents and conditions: (a) NaOH aqueous solution, r.t., 3 h, then HCl acidification; (b) SOCl_2_, toluene, reflux, 3 h; (c) 1,2,3,4-tetrahydroquinoline, pyridine, CH_2_Cl_2_, r.t., 1 h

General synthetic procedure of title compounds **10a–10p** is shown in Scheme 3. The saponification of the ester intermediate **3** afforded the substituted-1*H*-pyrazole-5-carboxylic acid **8** [33]. The title compounds **10** were prepared by the amidation of compounds **9** and 1,2,3,4-tetrahydroquinoline (THQ) [34].

The structures of all the title compounds were confirmed by ^1^H NMR, ^13^C NMR, IR spectra and HRMS or elemental analysis and the relevant data could be found in the Additional file 1. Compound **10a** was taken as an example to analyze the ^1^H NMR spectra data. Four protons of the benzene ring were observed at *δ* 7.18–6.87. A single peak at *δ* 5.76 was due to the proton at the 4-position of the parazole ring. Two protons at the 2-position of THQ were observed at *δ* 3.90 with *J* = 6.5 Hz as a triple peak, and the other triple peak at *δ* 2.82 with *J* = 6.6 Hz was due to the protons at the 4-position of THQ. Two protons at the 3-position of THQ was showed at *δ* 2.03 with *J* = 6.6 Hz as pentaploid peaks. The chemical shifts as single peaks were observed at *δ* 3.87 and 2.15 due to the protons of N-CH_3_ and CH_3_ at the 3-position of the parazole ring respectively.

**Fig. 2:**
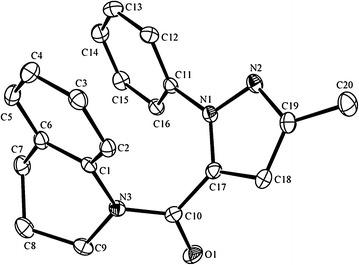
The X-ray crystal structure of **10g**

In order to further confirm the structure of the title compounds, a single crystal of **10g** (R^1^ = Ph, R^2^ = Me) was prepared for the X-ray diffraction. The single crystal was obtained by slow evaporation of a solution of compound **10g** in ethyl acetate at room temperature. As shown in Fig. 2, the crystal data for **10g**: orthorhombic, space group P2_1_2_1_2_1_ (no. 19), *a* = 8.3512(9) Å, *b* = 12.5600(13) Å, *c* = 15.3638(16) Å, *V* = 1611.5(3) Å^3^, *Z* = 4, *T* = 180.01(10) K, μ(Mo Kα) = 0.083 mm^−1^, *Dcalc* = 1.308 g/mm^3^, 5965 reflections measured (5.858 ≤ 2Θ ≤ 52.042), 3141 unique (*R*_int_ = 0.0292) which were used in all calculations. The final *R*_1_ was 0.0369 (I > 2σ(I)) and *wR*_2_ was 0.0852. Crystallographic data have been deposited with the Cambridge Crystallographic Data Centre as supplementary publication number CCDC 1441750. For more information on crystal data, see the Additional files 2 and 3.

### Biological activity

The in vitro fungicidal activities of all the title compounds have been determined against seven pathogenic fungi at the concentration of 50 μg/mL, and the mycelium growth rate method was used [35, 36]. Pyraclostrobin (Fig. 1) was assessed as a positive control. The bioassay results, illustrated in Table 1, indicated that the title compounds exhibited obvious fungicidal activities. Most of them displayed satisfied activities against *V. mali*, *S. sclerotiorum* and *G. graminis* var. *tritici*. Particularly, compounds **10d**, **10e**, **10i** and **10j** showed inhibitory activities of more than 85 % against *V. mali*. Compounds **10d**, **10e**, **10f, 10h**, **10i, 10j** and **10l** also demonstrated good activities against *S. sclerotiorum.* Especially, five title compounds (**10d**, **10e**, **10h**, **10i** and **10j**) exhibited striking activities against *G. graminis* var. *tritici*, with more than 90 % inhibition rates.

**Table 1:** Fungicidal activities of title compounds against seven kinds of pathogenic fungi

Compd.	R^1^	R^2^	Fungicidal activity (%)/50 μg/mL						
			*P. a*	*R. s*	*V. m*	*S. s*	*B. c*	*F. m*	*G. g. t*
**10a**	Me	Me	5.2	19.7	17.8	33.6	6.9	11.8	4.5
**10b**	Me	Et	12.9	30.7	14.4	40.1	5.7	15.8	31.7
**10c**	Me	*i*-Pr	12.1	40.6	53.0	72.8	20.8	17.0	8.9
**10d**	Me	Ph	35.1	62.2	91.9	92.6	74.1	49.7	100
**10e**	Me	4-OMePh	25.4	63.4	91.5	84.8	61.8	48.5	100
**10f**	Me	4-ClPh	15.3	54.7	57.6	85.3	52.3	27.4	35.7
**10g**	Ph	Me	30.6	26.8	23.3	48.4	28.8	22.6	79.0
**10h**	Ph	Et	40.3	39.4	65.7	84.3	66.2	48.5	99.1
**10i**	Ph	*n*-Pr	53.6	61.0	86.4	97.2	78.9	54.5	96.1
**10j**	Ph	*i*-Pr	50.4	56.7	86.0	88.0	79.3	50.9	90.1
**10k**	Ph	Ph	12.1	33.5	47.5	72.8	37.1	36.2	87.1
**10l**	2-ClPh	Me	20.2	19.7	49.6	88.5	35.1	21.0	78.6
**10m**	2-ClPh	4-ClPh	4.8	22.4	36.9	47.9	36.7	17.8	76.4
**10n**	*t*-Bu	Me	17.7	24.8	24.6	32.7	24.0	17.0	26.3
**10o**	3-ClPy	Cl	24.2	26.8	39.8	45.6	47.9	27.0	65.7
**10p**	3-ClPy	Br	38.7	39.0	56.8	59.9	42.7	28.6	72.6
Pyraclostrobin	–	–	47.4	100	89.0	100	84.5	78.5	100

*P. a: Pythium aphanidermatum, R. s: Rhizoctonia solani, V. m: Valsa mali, S. s: Sclerotinia sclerotiorum, B. c: Botrytis cinerea, F. m: Fusarium moniliforme, G. g. t: Gaeumannomyces graminis* var. *tritici*

Primary structure activity relationships (SAR) revealed that the substituents played an important role in fungicidal activities. (1) When substituent R^1^ was methyl, compounds with R^2^ as (substituted) phenyl exhibited better activities than those with R^2^ as alkyl (**10d**, **10e**, **10f** > **10a**, **10b**, **10c**). (2) When R^1^ was phenyl, the fungicidal activities increased with the increase of the carbon number in the alkyl chain of the R^2^ moiety (**10g** < **10h** < **10i** ≈ **10j**). However, fungicidal activities decreased dramatically when R^1^ and R^2^ were both phenyl (**10k**). (3) It was not beneficial to increase their fungicidal activities when R^1^ was substituted pyridyl (**10o** and **10p**).

In particular, compounds **10d** (R^1^ = Me, R^2^ = Ph), **10e** (R^1^ = Me, R^2^ = 4-OMePh), **10i** (R^1^ = Ph, R^2^ = *n*-Pr) and **10j** (R^1^ = Ph, R^2^ = *i*-Pr) exhibited good activities against *V. mali, S. sclerotiorum* and *G. graminis* var. *tritici* with inhibition rates of more than 80 %. Compounds **10d** and **10e** showed comparable activities against *V. mali* and *G. graminis* var. *tritici* with the commercial fungicide pyraclostrobin.

In the further study, fungicidal activities against *G. graminis* var. *tritici* of compounds **10d**, **10e**, **10h**, **10i** and **10j** were evaluated at lower concentrations (Table 2). Obviously, the result revealed a dosage-dependent relationship. Compounds **10d** and **10e** still exhibited satisfied activities with the inhibition rates of 100 % and 94.0 % at the concentration of 16.7 μg/mL, respectively, which is comparable to that of the positive control using pyraclostrobin. Unfortunately, their fungicidal activities decreased dramatically at the concentration of 11.1 μg/mL.

**Table 2:** Dosage-dependent in vitro fungicidal activities of **10d**, **10e**, **10h**, **10i**, **10j** and pyraclostrobin against *G. graminis* var. *tritici*

Compd.	Inhibition rate (%) at different concentrations (μg/mL)				
	50.0	25.0	16.7	11.1	2.2
**10d**	100	100	100	65.7	1.0
**10e**	100	100	94.0	47.7	−8.4
**10h**	99.1	88.9	57.1	37.0	6.1
**10i**	96.1	88.0	74.3	63.1	34.9
**10j**	90.1	51.1	46.0	37.9	21.1
Pyraclostrobin	100	100	100	100	92.7

## Experimental

### Chemistry

Melting points of all compounds were determined on an X-4 binocular microscope (Fukai Instrument Co., Beijing, China) without calibration. NMR spectra were acquired with a Bruker 300 MHz spectrometer with CDCl_3_ as the solvent and TMS as the internal standard. Chemical shifts are reported in *δ* (parts per million) values. High resolution mass spectrometry (HRMS) data were obtained on an FTICR-MS Varian 7.0T FTICR-MS instrument. Elemental analysis was carried out on a Vario EL III elemental analyzer. All the reagents were obtained commercially and used without further purification. Column chromatography purification was carried out by using silica gel. The synthesis of intermediates and title compounds can be found in the Additional file 1.

### Antifungal biological assay

All the target compounds have been evaluated for their in vitro fungicidal activities against seven pathogenic fungi, using mycelium growth rate method according to the literature [35, 36]. Fungi tested in this article included *Pythium aphanidermatum, Rhizoctonia solani, Valsa mali, Sclerotinia sclerotiorum, Botrytis cinerea, Fusarium moniliforme* and G*aeumannomyces graminis* var. *tritici*. Dimethyl sulfoxide (DMSO) in sterile distilled water served as the control. Pyraclostrobin (Fig. 1) containing pyrazole structure (Fig. 1) as the commercial fungicide, was assessed under the same conditions as a positive control. In the preparation, every compound (10 mg) was weighted accurately and dissolved in 1 mL DMSO, and then it was mixed with 200 mL potato dextrose agar (PDA). As a consequence, they were tested at a concentration of 50 μg/mL. In order to get new mycelium for antifungal assay, all fungal species were incubated in PDA at 25 ± 1 °C for 1–7 days vary from different fungi. Mycelia dishes were cut with a 5 mm in diameter hole punch from the prepared edge of culture medium. One of them was picked up with a sterilized inoculation needle, and then inoculated in the center of the PDA plate aseptically. Every treatment repeated three times, and they were incubated at 25 ± 1 °C for 1–7 days vary from different fungi. All the above was completed in a bioclean environment. The hypha diameter was measured by a ruler, and the data were statistically analyzed. The inhibition rate of the title compounds on the fungi was calculated by the following formula:

I (%) = [(C − T)/(C − 5)] × 100, where I is the inhibition rate, C represents the diameter (mm) of fungal growth on untreated PDA, and T represents the diameter (mm) of fungi on treated PDA.

## Conclusion

In summary, a series of pyrazole derivatives containing 1,2,3,4-tetrahydroquinoline were synthesized and their structures were confirmed by ^1^H NMR, ^13^C NMR, IR and HRMS or elemental analysis. The crystal structure of compound **10g** was determined by X-ray diffraction. Bioassay results indicated that all the title compounds exhibited good fungicidal activities. And the substituents played an important role in fungicidal activities. In particular, compounds **10d** and **10e** with simple structures showed comparable activities against *G. graminis* var. *tritici* to the commercial fungicide pyraclostrobin even at the concentration 16.7 μg/mL. These two compounds could be valuable leads for further studies.

## Additional files

10.1186/s13065-016-0186-8 The experimental procedures of intermediates **3**, **5**, **6**, **7**, **8**, **9** and title compounds **10**, and the data of ^1^H NMR, ^13^C NMR, IR and HRMS or elemental analysis of target compounds **10**.
10.1186/s13065-016-0186-8 Structure description of the compound **10g**. Which includes bond lengths and bond angles.
10.1186/s13065-016-0186-8 Structural information (CIF) for Compound **10g**.
