# Supplementary material for: Synthesis and fungicidal activity of pyrazole derivatives containing 1,2,3,4-tetrahydroquinoline
Source: Chem Cent J. 2016 Jul 4;10:40. doi: 10.1186/s13065-016-0186-8 (PMC4932680; doi:10.1186/s13065-016-0186-8)
Supplement: Supplementary file 1 — 10.1186/s13065-016-0186-8 The experimental procedures of intermediates 3, 5, 6, 7, 8, 9 and title compounds 10, and the data of 1H NMR, 13C NMR, IR and HRMS or elemental analysis of target compounds 10. [file 13065_2016_186_MOESM1_ESM.docx]

Additional file 1

**Synthesis and fungicidal activity of pyrazole derivatives containing 1,2,3,4-tetrahydroquinoline**

Peng Lei ^1^, Xuebo Zhang ^1^, Yan Xu ^1^, Gaofei Xu ^1^, Xili Liu ^2^, Xinling Yang ^1^, Xiaohe Zhang ^1^, Yun Ling ^1 ^[[1]](#footnote-1)^*^

*^1^ Department of Applied Chemistry, College of Science, China Agricultural University, Beijing 100193, China*

*^2^ Department of Plant Pathology, China Agricultural University, Beijing 100193, China*

**Supporting Information**

**Table of contents**

[Synthetic route S1](#_Toc449831312)

[Experimental Procedure S2](#_Toc449831313)

[References S4](#_Toc449831314)

[Analytical data of compounds 10a−10p S5](#_Toc449831320)

[NMR Spectra S9](#_Toc449831321)

**Synthetic route**

**Scheme 1** Synthetic route of intermediates **3a – 3n**.

**Scheme 2** Synthetic route of intermediates **3o – 3p**.

**Scheme 3** Synthetic route of the target compounds **10**.

# Experimental Procedure

**General synthetic procedure for compounds 3a – 3n according to the literature [1].**

Sodium (0.06 mol) cut in pieces was added slowly to 100 mL absolute ethanol. Until completion of the reaction, diethyl oxalate (0.05 mol) was added and stirred for 10 min. Then substituted ketone **1** (0.05 mol) was added dropwise to the mixture and stirred at room temperature for 2h. Glacial acetic acid (0.06 mol) was added to the resultant mixture and stirred for 30 min. Substituted hydrazine (0.06 mol) was added slowly to the reaction mixture, and then the mixture was stirred at room temperature for overnight. The final mixture was concentrated under reduced pressure, and 100 mL water was added, the crude product **3a – 3n** was obtained through extraction with dichloromethane (50 mL×2), drying with anhydrous sodium sulfate and desolventizing, then it was purified by column chromatography with silica gel.

**Synthesis of intermediate 5 according to the literature [2-3].**

A mixture solution of 2,3-dichloropyridine **4** (11.84 g, 0.080 mol) and hydrazine hydrate (80%) (48 g, 0.76 mol) was heated to reflux for 5 h, and then cooled to room temperature. The crude product precipitated was collected by filtration, washed with ethanol, and dried to afford white crystals **5**.

**Synthesis of intermediate 6 according to the literature [2-3].**

Sodium (1.932 g, 0.084 mol) cut in pieces was added slowly to 55 mL absolute ethanol. Until completion of the reaction, the mixture was heated to reflux and (3-chloropyridin-2-yl)hydrazine (**5**) (10.88 g, 0.076 mol) was added. Then Diethyl maleate (14.46 g, 0.084 mol) was added dropwise to the mixture after it was refluxed for 10 min. The reaction mixture was stirred at reflux for another 30 min. After the mixture was cooled to 65 ℃, treated with glacial acetic acid (9.60 g, 0.16 mol), and concentrated in vacuo. The residue was treated with water (60 mL) and filtered. The resulting solid was added to 70% ethanol and stirred thoroughly. The intermediate **6** was isolated via filtration, washed with 50% ethanol, dried to give a white solid.

**General synthetic procedure for intermediate 7 according to the literature [2-3].**

A solution of phosphorus oxychloride or phosphorus oxybromide (0.0097 mol) in 5 mL acetonitrile was added dropwise to a solution of **6** (2.18 g, 0.0081 mol) in acetonitrile (21 mL). The mixture was heated to reflux for 4h, then concentrated. The concentrated reaction mixture was slowly poured into saturated aq. sodium carbonate forming a weak alkaline solution, and stirred vigorously for 30 min. Then the crude intermediate **7** was obtained through extraction with dichloromethane (40 mL×2), drying with anhydrous sodium sulfate and desolventizing, and then it was purified by column chromatography with silica gel.

**General synthetic procedure for intermediate 3o-3p according to the literature [2-3].**

A solution of **7** (0.0068 mol), 98% sulfuric acid (0.8 mL) in acetonitrile (30 mL) was stirred for 10 min. Then potassium persulfate (2.76 g, 0.0102 mol) was added to the mixture and heated to reflux for 4h. After the mixture was cooled to 50 ℃, filtered, washed with acetonitrile. The filtrate was concentrated under reduced pressure and added slowly to 30 mL water under stirring for 30 min. The solid was collected by filtration, washed with 25% acetonitrile and water, and then dried to get the intermediates **3o-3p**.

**General synthetic procedure for intermediate 8 according to the literature [4].**

A solution of intermediate **3** (0.01 mol), 10 mL methanol and 8 mL aqueous sodium hydroxide solution (1.5 mol•L^-1^) was stirred at room temperature for 3 h. The mixture solution was concentrated under reduced pressure, and the residue was dilute with 50 mL water. Washed with dichloromethane to remove organic impurities, the aqueous phase was acidified to pH=2 using concentrated hydrochloric acid, filtered and washed with water to afford the intermediate **8**.

**General synthetic procedure for title compounds 10 according to the literature [5].**

A solution of thionyl chloride (0.007 mol) was added dropwise to **8** (0.005 mol) in toluene (5 mL). The mixture was heated to reflux for 3 h. Excess thionyl chloride and toluene was removed in vacuo to give the crude intermediate **9**.

A solution of the crude intermediate **9** in 5 mL dichloromethane was added dropwise to a solution of 1,2,3,4-tetrahydroquinoline (0.005 mol) and pyridine (0.005 mol) in 5 mL dichloromethane. The reaction mixture was stirred at room temperature and monitored by TLC. After the mixture washed with water, the organic phase was concentrated under reduced pressure, and then purified by column chromatography eluting with ethyl acetate/petroleum ether to obtain the pure product **10**.

# References

# 1. Liu M, Liu DK, Liu Y, Xu WR, Zhang SJ, Zhang CY, Tang LD: Preparation of pyrazolo[1,5-a]pyridine derivatives as antitumor and/or antiviral agents. CN 101544634A, 2009.09.30.

# 2. Xu JY, Dong WL, Xiong LX, Li YX, Li ZM: Design, synthesis and biological activities of novel amides (sulfonamides) containing N-pyridylpyrazole. *Chin J Chem* 2009, 27:2007-2012.

# 3. Zhao Y, Li YQ, Xiong LX, Wang HX, Li ZM: Design, synthesis and biological activities of novel anthranilic diamide insecticide containing trifluoroethyl ether. *Chin J Chem* 2012, 30:1748-1758.

# 4. Zhu HW, Wang BL, Zhang XL, Xiong LX, Yu SJ, Li ZM: Syntheses and biological activities of novel 3-bromo-1-(3-chloropyridin-2-yl)-*N*-hydroxy-N-aryl- 1*H*-pyrazole-5-carboxamides. *Chem Res Chin Univ* 2014, 30:409-414.

# 5. Liu SH, Ling Y, Yang XL: Synthesis, bioactivities and crystal structure of (Z)- N-(3-((2-(4-chlorophenyl)-oxazol-4-yl)methyl)thiazolidin-2-ylidene)cyanamide. *Chinese J Struct Chem* 2013, 32:931-935.

# Analytical data of compounds 10a−10p

*(3,4-dihydroquinolin-1(2H)-yl)(1,3-dimethyl-1H-pyrazol-5-yl)methanone* (**10a**): yellow oil; yield 73.7%; ^1^H NMR (300 MHz, CDCl_3_) δ 7.17 (d, *J* = 7.4 Hz, 1H), 7.08 (td, *J* = 7.4, 1.3 Hz, 1H), 7.00 (t, *J* = 7.6 Hz, 1H), 6.89 (d, *J* = 7.8 Hz, 1H), 5.76 (s, 1H), 3.90 (t, *J* = 6.5 Hz, 2H), 3.87 (s, 3H), 2.82 (t, *J* = 6.6 Hz, 2H), 2.15 (s, 3H), 2.03 (p, *J* = 6.6 Hz, 2H); ^13^C NMR (75 MHz, CDCl_3_) δ 161.03, 146.37, 138.02, 136.72, 131.35, 128.14, 125.67, 125.00, 124.18, 107.34, 44.07, 37.57, 26.43, 23.69, 12.88; IR (KBr): *ν* = 3029, 2947, 2890, 1643, 1581, 1493, 1447, 759 cm^-1^; HRMS Calcd. for C_15_H_18_N_3_O [M+H]^+^: 256.1444, Found: 256.1440.

*(3-ethyl-1-methyl-1H-pyrazol-5-yl)(3,4-dihydroquinolin-1(2H)-yl)methanone* (**10b**): yellow oil; yield 71.3%; ^1^H NMR (300 MHz, CDCl_3_) δ 7.18 – 7.14 (m, 1H), 7.07 (td, *J* = 7.4, 1.4 Hz, 1H), 7.01 – 6.96 (m, 1H), 6.88 (d, *J* = 7.9 Hz, 1H), 5.77 (s, 1H), 3.93 – 3.88 (m, 5H), 2.82 (t, *J* = 6.6 Hz, 2H), 2.53 (q, *J* = 7.6 Hz, 2H), 2.04 (p, *J* = 6.6 Hz, 2H), 1.12 (t, *J* = 7.6 Hz, 3H); ^13^C NMR (75 MHz, CDCl_3_) δ 161.11, 152.53, 138.01, 136.53, 131.33, 128.13, 125.59, 124.98, 124.21, 105.99, 44.07, 37.59, 26.45, 23.70, 20.81, 13.49; IR (KBr): *ν* = 3030, 2964, 2939, 2888, 1642, 1581, 1493, 1468, 1440, 758 cm^-1^; HRMS Calcd. for C_16_H_20_N_3_O [M+H]^+^: 270.1601, Found: 270.1605.

*(3,4-dihydroquinolin-1(2H)-yl)(3-isopropyl-1-methyl-1H-pyrazol-5-yl)methanone* (**10c**): yellow oil; yield 77.2%; ^1^H NMR (300 MHz, CDCl_3_) δ 7.17 (d, *J* = 7.2 Hz, 1H), 7.07 (td, *J* = 7.4, 1.3 Hz, 1H), 6.98 (t, *J* = 7.7 Hz, 1H), 6.87 (d, *J* = 8.0 Hz, 1H), 5.75 (s, 1H), 3.91 (t, *J* = 6.5 Hz, 5H), 2.91 – 2.80 (m, 3H), 2.04 (p, *J* = 6.5 Hz, 2H), 1.14 (d, *J* = 6.9 Hz, 6H); ^13^C NMR (75 MHz, CDCl_3_) δ 161.20, 157.07, 138.03, 136.31, 131.32, 128.12, 125.50, 124.96, 124.26, 104.68, 44.07, 37.61, 27.24, 26.47, 23.72, 22.39; IR (KBr): *ν* = 3030, 2960, 2889, 2870, 1642, 1604, 1582, 1493, 1474, 1454, 757 cm^-1^; HRMS Calcd. for C_17_H_22_N_3_O [M+H]^+^: 284.1757, Found: 284.1754.

*(3,4-dihydroquinolin-1(2H)-yl)(1-methyl-3-phenyl-1H-pyrazol-5-yl)methanone* (**10d**): faint yellow solid; yield 69.2%; m.p. 90～91℃; ^1^H NMR (300 MHz, CDCl_3_) δ 7.66 – 7.62 (m, 2H), 7.38 – 7.27 (m, 3H), 7.20 (d, *J* = 6.9 Hz, 1H), 7.09 (td, *J* = 7.4, 1.5 Hz, 1H), 7.02 – 6.90 (m, 2H), 6.24 (s, 1H), 3.99 (s, 3H), 3.95 (t, *J* = 6.5 Hz, 2H), 2.85 (t, *J* = 6.6 Hz, 2H), 2.07 (p, *J* = 6.5 Hz, 2H); ^13^C NMR (75 MHz, CDCl_3_) δ 160.74, 149.32, 137.87, 137.46, 132.41, 131.53, 128.29, 127.50, 125.82, 125.24, 125.15, 124.25, 104.97, 44.13, 38.21, 26.51, 23.74; IR (KBr): *ν* = 3037, 2967, 2926, 2894, 2870, 1641, 1606, 1583, 1493, 1445, 760 cm^-1^; Anal. calcd for C_20_H_19_N_3_O: C 75.69, H 6.03, N 13.24; found C 75.81, H 6.00, N 13.26.

*(3,4-dihydroquinolin-1(2H)-yl)(3-(4-methoxyphenyl)-1-methyl-1H-pyrazol-5-yl)methanone* (**10e**): yellow solid; yield 91.5%; m.p. 117～119℃; ^1^H NMR (300 MHz, CDCl_3_) δ 7.59 – 7.54 (m, 2H), 7.19 (d, *J* = 7.3 Hz, 1H), 7.08 (td, *J* = 7.4, 1.4 Hz, 1H), 7.01 – 6.86 (m, 4H), 6.18 (s, 1H), 3.94 (t, *J* = 6.5 Hz, 5H), 3.81 (s, 3H), 2.84 (t, *J* = 6.6 Hz, 2H), 2.06 (p, *J* = 6.6 Hz, 2H); ^13^C NMR (75 MHz, CDCl_3_) δ 160.81, 159.07, 149.18, 137.88, 137.37, 131.46, 128.25, 126.40, 125.79, 125.18, 124.24, 113.66, 104.43, 54.95, 44.16, 38.07, 26.50, 23.72; IR (KBr): *ν* = 3031, 2937, 2837, 1651, 1602, 1580, 1494, 1448, 814, 759 cm^-1^; Anal. calcd for C_21_H_21_N_3_O_2_: C 72.60, H 6.09, N 12.10; found C 72.80, H 6.11, N 12.03.

*(3-(4-chlorophenyl)-1-methyl-1H-pyrazol-5-yl)(3,4-dihydroquinolin-1(2H)-yl)methanone* (**10f**): yellow solid; yield 90.4%; m.p. 119～121℃; ^1^H NMR (300 MHz, CDCl_3_) δ 7.59 – 7.54 (m, 2H), 7.33 – 7.26 (m, 2H), 7.20 (d, *J* = 7.2 Hz, 1H), 7.09 (td, *J* = 7.5, 1.3 Hz, 1H), 6.99 (t, *J* = 7.6 Hz, 1H), 6.89 (d, *J* = 8.3 Hz, 1H), 6.20 (s, 1H), 3.98 (s, 3H), 3.94 (t, *J* = 6.5 Hz, 2H), 2.85 (t, *J* = 6.7 Hz, 2H), 2.07 (p, *J* = 6.6 Hz, 2H); ^13^C NMR (75 MHz, CDCl_3_) δ 160.54, 148.19, 137.79, 137.65, 133.20, 131.59, 130.93, 128.43, 128.29, 126.39, 125.80, 125.31, 124.23, 104.88, 44.10, 38.25, 26.50, 23.72; IR (KBr): *ν* = 3027, 2953, 2884, 2846, 1640, 1604, 1579, 1489, 1462, 819, 763 cm^-1^; Anal. calcd for C_20_H_18_ClN_3_O: C 68.28, H 5.16, N 11.94; found C 68.23, H 5.16, N 11.75.

*(3,4-dihydroquinolin-1(2H)-yl)(3-methyl-1-phenyl-1H-pyrazol-5-yl)methanone* (**10g**): yellow solid;yield 99.7%;m.p. 119～121℃; ^1^H NMR (300 MHz, CDCl_3_) δ 7.22 – 7.17 (m, 3H), 6.96 – 6.88 (m, 4H), 6.79 (s, 1H), 6.56 (s, 2H), 3.76 (s, 2H), 2.35 (s, 3H), 2.14 (s, 2H), 1.75 (p, *J* = 6.6 Hz, 2H); ^13^C NMR (75 MHz, CDCl_3_) δ 160.93, 149.30, 138.92, 137.58, 136.78, 131.95, 128.37, 127.76, 127.16, 125.58, 124.57, 122.88, 122.57, 109.66, 43.46, 25.85, 22.54, 13.19; IR (KBr): *ν* = 3044, 2959, 2926, 2888, 2868, 1632, 1600, 1580, 1491, 1462, 1451, 752 cm^-1^; Anal. calcd for C_20_H_19_N_3_O: C 75.69, H 6.03, N 13.24; found C 75.89, H 5.86, N 13.40.

*(3-ethyl-1-phenyl-1H-pyrazol-5-yl)(3,4-dihydroquinolin-1(2H)-yl)methanone* (**10h**): yellow oil; yield 78.2%; ^1^H NMR (300 MHz, CDCl_3_) δ 7.25 – 6.59 (m, 10H), 3.76 (s, 2H), 2.72 (q, *J* = 7.6 Hz, 2H), 2.14 (s, 2H), 1.75 (p, *J* = 6.6 Hz, 2H), 1.29 (t, *J* = 7.6 Hz, 3H); ^13^C NMR (75 MHz, CDCl_3_) δ 161.09, 155.33, 139.04, 137.44, 136.89, 131.86, 128.34, 127.75, 127.10, 126.67, 125.51, 124.53, 122.95, 122.58, 108.21, 43.49, 25.86, 22.57, 21.05, 13.52; IR (KBr): *ν* = 3039, 2967, 2947, 2876, 1639, 1597, 1579, 1493, 1468, 1443, 756 cm^-1^; HRMS Calcd. for C_21_H_22_N_3_O [M+H]^+^: 332.1757, Found: 332.1761.

*(3,4-dihydroquinolin-1(2H)-yl)(1-phenyl-3-propyl-1H-pyrazol-5-yl)methanone* (**10i**): yellow oil; yield 71.1%; ^1^H NMR (300 MHz, CDCl_3_) δ 7.22 - 7.16 (m, 3H), 6.95 – 6.76 (m, 5H), 6.57 (s, 2H), 3.76 (s, 2H), 2.66 (t, *J* = 7.5 Hz, 2H), 2.13 (s, 2H), 1.79 – 1.66 (m, 4H), 0.99 (t, *J* = 7.4 Hz, 3H); ^13^C NMR (75 MHz, CDCl_3_) δ 161.12, 153.88, 139.03, 137.37, 136.90, 131.93, 128.34, 127.73, 127.08, 125.48, 124.53, 122.94, 122.59, 108.72, 43.42, 29.72, 25.86, 22.57, 22.47, 13.41; IR (KBr): *ν* = 3029, 2957, 2931, 2872, 1642, 1599, 1581, 1493, 1464, 1445, 759 cm^-1^; HRMS Calcd. for C_22_H_24_N_3_O [M+H]^+^: 346.1914, Found: 346.1912.

*(3,4-dihydroquinolin-1(2H)-yl)(3-isopropyl-1-phenyl-1H-pyrazol-5-yl)methanone* (**10j**): yellow oil; yield 72.6%; ^1^H NMR (300 MHz, CDCl_3_) δ 7.23 – 7.16 (m, 3H), 6.99 – 6.77 (m, 5H), 6.60 (s, 2H), 3.76 (s, 2H), 3.05 (dt, *J* = 13.9, 7.0 Hz, 1H), 2.16 (s, 2H), 1.75 (p, *J* = 6.6 Hz, 2H), 1.31 (d, *J* = 6.9 Hz, 6H); ^13^C NMR (75 MHz, CDCl_3_) δ 161.22, 159.82, 139.10, 137.20, 136.90, 131.88, 128.34, 127.74, 127.05, 125.42, 124.51, 122.97, 122.56, 106.82, 43.44, 27.50, 25.87, 22.58, 22.49; IR (KBr): *ν* = 2959, 2931, 2870, 1641, 1599, 1582, 1493, 1475, 1461, 759 cm^-1^; HRMS Calcd. for C_22_H_24_N_3_O [M+H]^+^: 346.1914, Found: 346.1918.

*(3,4-dihydroquinolin-1(2H)-yl)(1,3-diphenyl-1H-pyrazol-5-yl)methanone* (**10k**): yellow oil; yield 68.6%; ^1^H NMR (300 MHz, CDCl_3_) δ 7.89 – 7.82 (m, 2H), 7.46 – 7.32 (m, 4H), 7.24 – 6.60 (m, 9H), 3.80 (s, 2H), 2.16 (s, 2H), 1.78 (p, *J* = 6.6 Hz, 2H); ^13^C NMR (75 MHz, CDCl_3_) δ 160.69, 151.80, 139.05, 138.37, 136.77, 132.10, 128.44, 128.34, 127.94, 127.84, 127.45, 125.71, 125.52, 124.69, 123.04, 122.65, 107.08, 43.59, 25.91, 22.57; IR (KBr): *ν* = 3043, 2947, 2842, 1639, 1597, 1581, 1493, 1458, 1437, 764 cm^-1^; HRMS Calcd. for C_25_H_22_N_3_O [M+H]^+^: 380.1757, Found: 380.1752.

*(1-(2-chlorophenyl)-3-methyl-1H-pyrazol-5-yl)(3,4-dihydroquinolin-1(2H)-yl)methanone* (**10l**): faint yellow solid; yield 100%; m.p. 147～149℃; ^1^H NMR (300 MHz, CDCl_3_) δ 7.38 (dd, *J* = 8.0, 1.4 Hz, 1H), 7.28 - 7.22 (m, 1H), 7.12 (td, *J* = 7.6, 1.2 Hz, 1H), 7.04 – 6.89 (m, 5H), 6.41 (s, 1H), 3.81 (s, 2H), 2.38 (s, 2H), 2.33 (s, 3H), 1.82 (p, *J* = 6.5 Hz, 2H); ^13^C NMR (75 MHz, CDCl_3_) δ 160.11, 149.21, 139.26, 137.51, 137.34, 131.53, 129.96, 129.29, 129.06, 128.30, 128.14, 126.98, 125.82, 124.73, 123.35, 109.48, 44.08, 26.08, 22.98, 13.20; IR (KBr): *ν* = 3039, 2956, 2939, 2884, 1639, 1582, 1494, 1455, 1446, 752 cm^-1^; Anal. calcd for C_20_H_18_ClN_3_O: C 68.28, H 5.16, N 11.94; found C 67.99, H 5.12, N 11.71.

*(1-(2-chlorophenyl)-3-(4-chlorophenyl)-1H-pyrazol-5-yl)(3,4-dihydroquinolin-1(2H)-yl)methanone* (**10m**): faint yellow solid; yield 89.7%; m.p. 81～83℃; ^1^H NMR (300 MHz, CDCl_3_) δ 7.77 – 7.72 (m, 2H), 7.44 – 7.27 (m, 4H), 7.17 (td, *J* = 7.6, 1.2 Hz, 1H), 7.06 – 6.87 (m, 6H), 3.84 (s, 2H), 2.41 (s, 2H), 1.85 (p, *J* = 6.5 Hz, 2H); ^13^C NMR (75 MHz, CDCl_3_) δ 159.66, 150.63, 140.20, 137.42, 137.30, 133.84, 131.64, 130.44, 130.05, 129.45, 129.40, 128.53, 128.40, 128.24, 127.06, 126.80, 125.94, 124.94, 123.39, 106.71, 44.13, 26.12, 23.00; IR (KBr): *ν* = 2950, 2886, 1642, 1604, 1581, 1492, 1451, 817, 754 cm^-1^; Anal. calcd for C_25_H_19_Cl_2_N_3_O: C 66.97, H 4.27, N 9.37; found C 66.97, H 4.53, N 9.16.

*(1-tert-butyl-3-methyl-1H-pyrazol-5-yl)(3,4-dihydroquinolin-1(2H)-yl)methanone* (**10n**): faint yellow solid; yield 94.2%; m.p. 105～106℃; ^1^H NMR (300 MHz, CDCl_3_) δ 7.49 (s, 1H), 7.17 – 7.04 (m, 3H), 5.89 (s, 1H), 3.75 (t, *J* = 5.7 Hz, 2H), 2.85 (t, *J* = 6.8 Hz, 2H), 2.21 (s, 3H), 2.00 (p, *J* = 6.4 Hz, 2H), 1.65 (s, 9H); ^13^C NMR (75 MHz, CDCl_3_) δ 163.55, 145.47, 137.59, 136.44, 129.98, 128.70, 125.65, 124.75, 123.65, 106.21, 60.66, 45.78, 29.72, 26.45, 23.20, 13.16; IR (KBr): *ν* = 2952, 2931, 2865, 1651, 1601, 1580, 1493, 1448, 769 cm^-1^; Anal. calcd for C_18_H_23_N_3_O: C 72.70, H 7.80, N 14.13; found C 72.60, H 7.67, N 14.05.

*(3-chloro-1-(3-chloropyridin-2-yl)-1H-pyrazol-5-yl)(3,4-dihydroquinolin-1(2H)-yl)methanone* (**10o**): faint yellow solid; yield 91.1%; m.p. 134～136℃; ^1^H NMR (300 MHz, CDCl_3_) δ 8.38 (dd, *J* = 4.7, 1.6 Hz, 1H), 7.84 (dd, *J* = 8.0, 1.6 Hz, 1H), 7.30 (dd, *J* = 8.0, 4.7 Hz, 1H), 7.17 (s, 1H), 7.09 – 7.02 (m, 3H), 6.17 (s, 1H), 3.83 (t, *J* = 6.5 Hz, 2H), 2.63 (t, *J* = 6.6 Hz, 2H), 1.97 (p, *J* = 6.6 Hz, 2H); ^13^C NMR (75 MHz, CDCl_3_) δ 158.77, 147.62, 146.20, 140.60, 140.10, 139.44, 137.24, 131.38, 128.07, 126.87, 126.19, 125.43, 124.50, 124.16, 108.27, 43.95, 26.28, 23.23; IR (KBr): *ν* = 3048, 2962, 2893, 1659, 1606, 1583, 1523, 1496, 1467, 768 cm^-1^; Anal. calcd for C_18_H_14_Cl_2_N_4_O: C 57.92, H 3.78, N 15.01; found C 58.02, H 3.91, N 14.88.

*(3-bromo-1-(3-chloropyridin-2-yl)-1H-pyrazol-5-yl)(3,4-dihydroquinolin-1(2H)-yl)methanone* (**10p**): white solid; yield 97.5%; m.p. 134～136℃; ^1^H NMR (300 MHz, CDCl_3_) δ 8.38 (dd, *J* = 4.7, 1.5 Hz, 1H), 7.84 (dd, *J* = 8.0, 1.6 Hz, 1H), 7.30 (dd, *J* = 8.0, 4.7 Hz, 1H), 7.17 (s, 1H), 7.11 – 6.96 (m, 3H), 6.26 (s, 1H), 3.83 (t, *J* = 6.5 Hz, 2H), 2.63 (t, *J* = 6.5 Hz, 2H), 1.97 (p, *J* = 6.6 Hz, 2H); ^13^C NMR (75 MHz, CDCl_3_) δ 158.63, 147.61, 146.23, 140.23, 139.43, 137.23, 131.35, 128.09, 127.18, 126.92, 126.18, 125.43, 124.55, 124.16, 111.54, 44.01, 26.28, 23.23; IR (KBr): *ν* = 3047, 2960, 2893, 1659, 1606, 1582, 1520, 1496, 1462, 768 cm^-1^; Anal. calcd for C_18_H_14_BrClN_4_O: C 51.76, H 3.38, N 13.41; found C 51.69, H 3.26, N 13.35.

**NMR Spectra**

**10a**


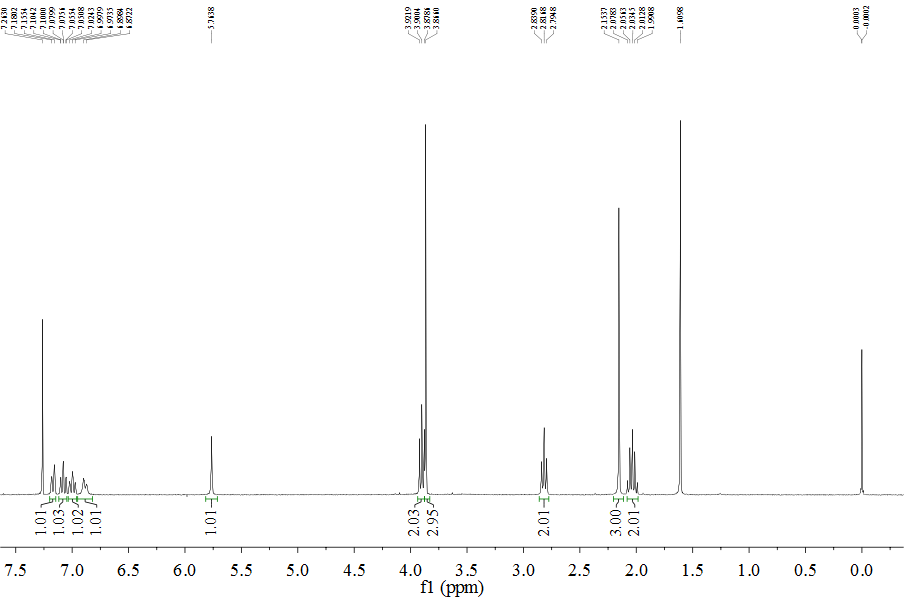

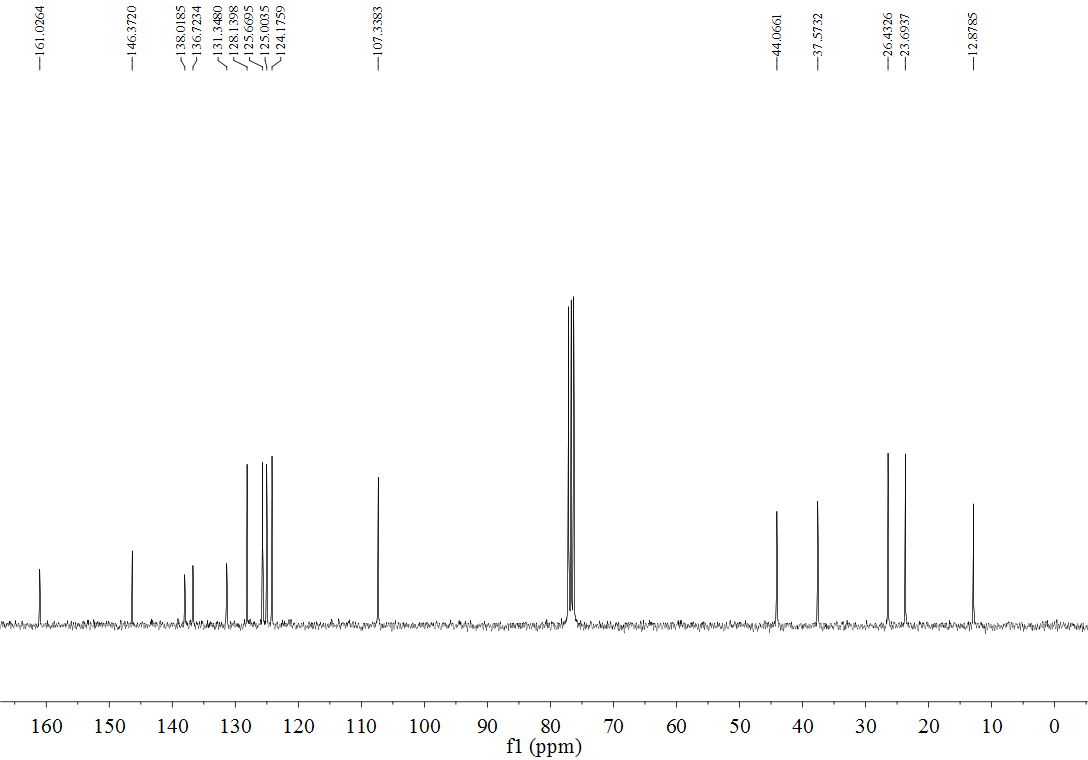

**10b**


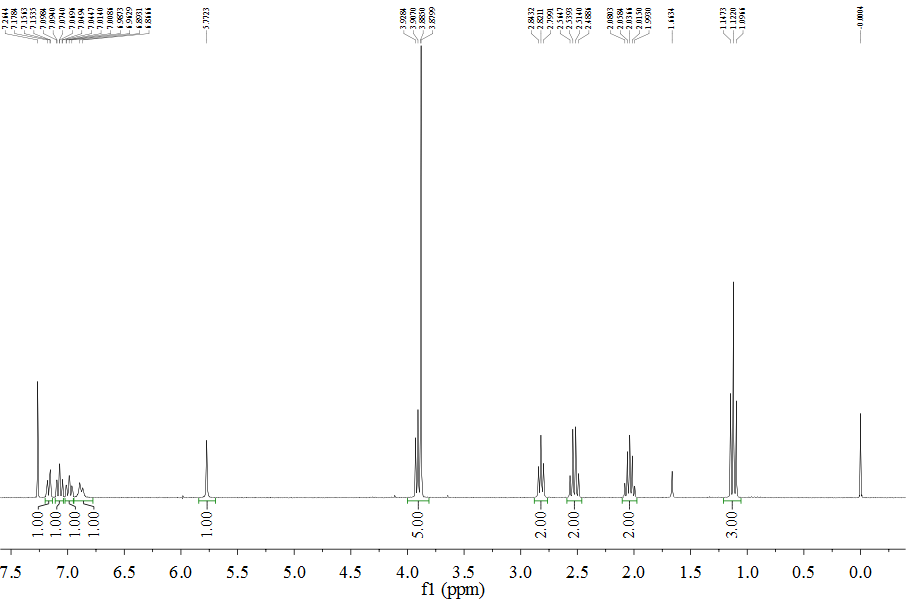

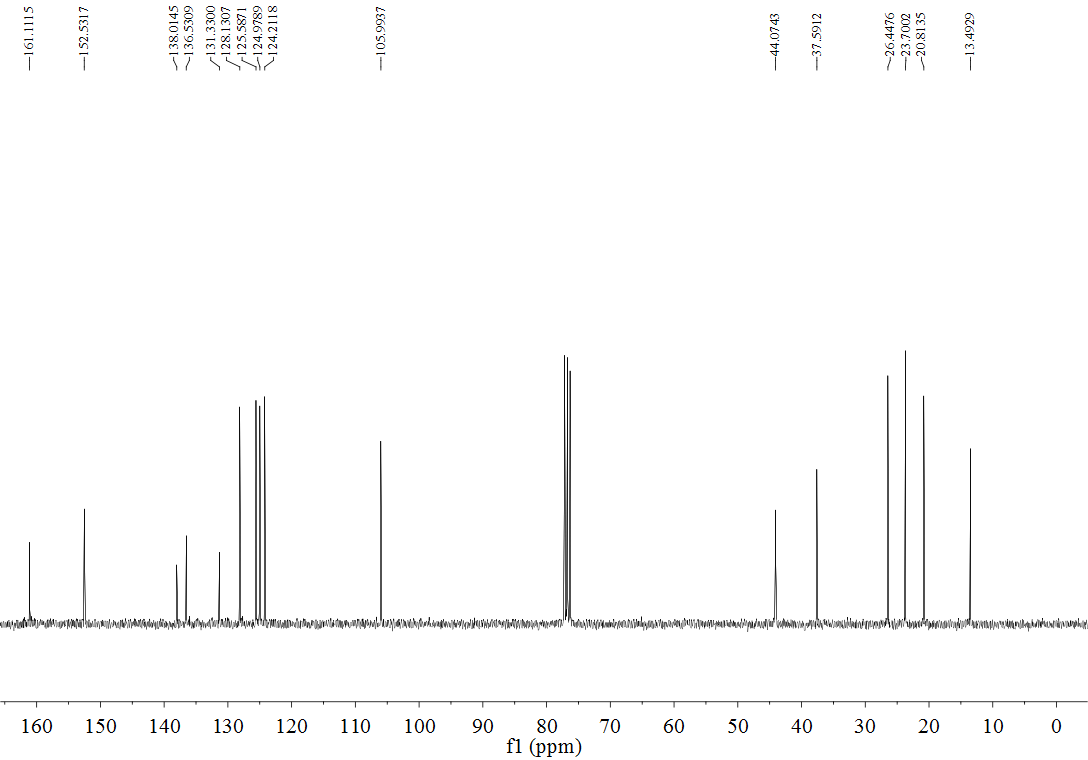

**10c**


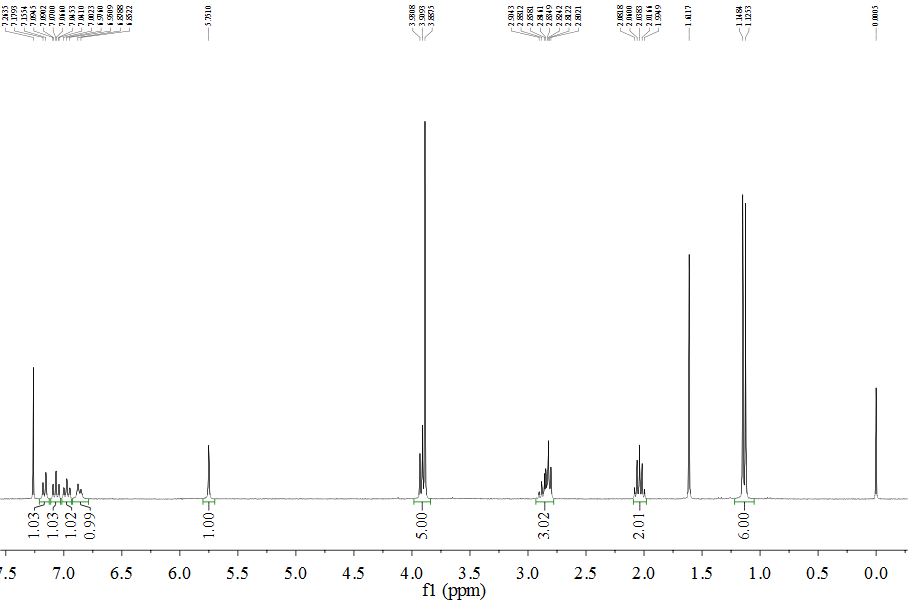

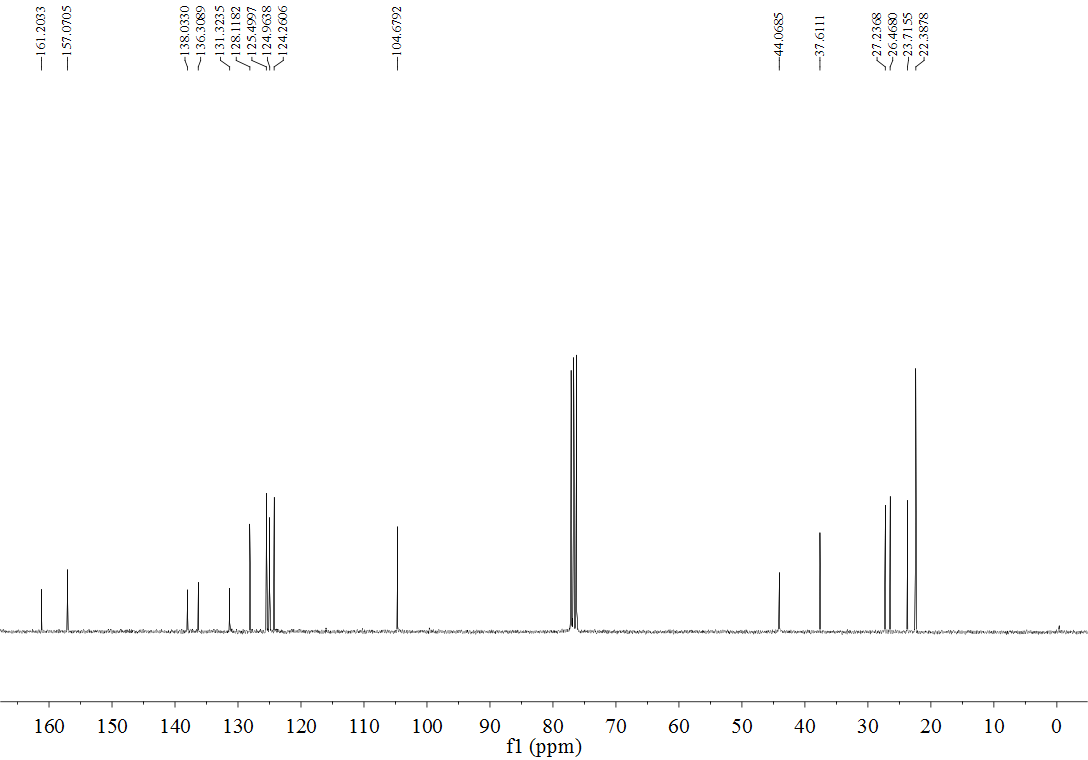

**10d**


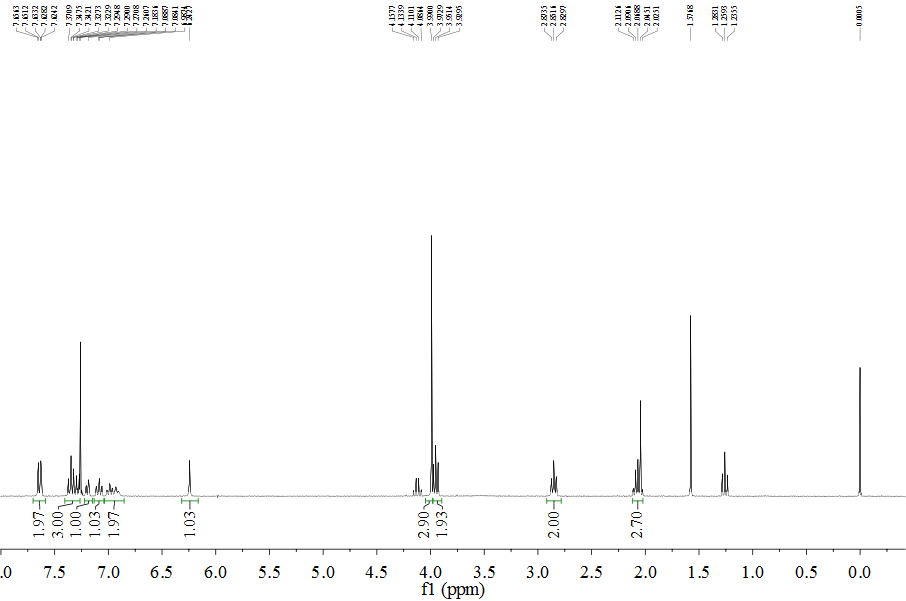

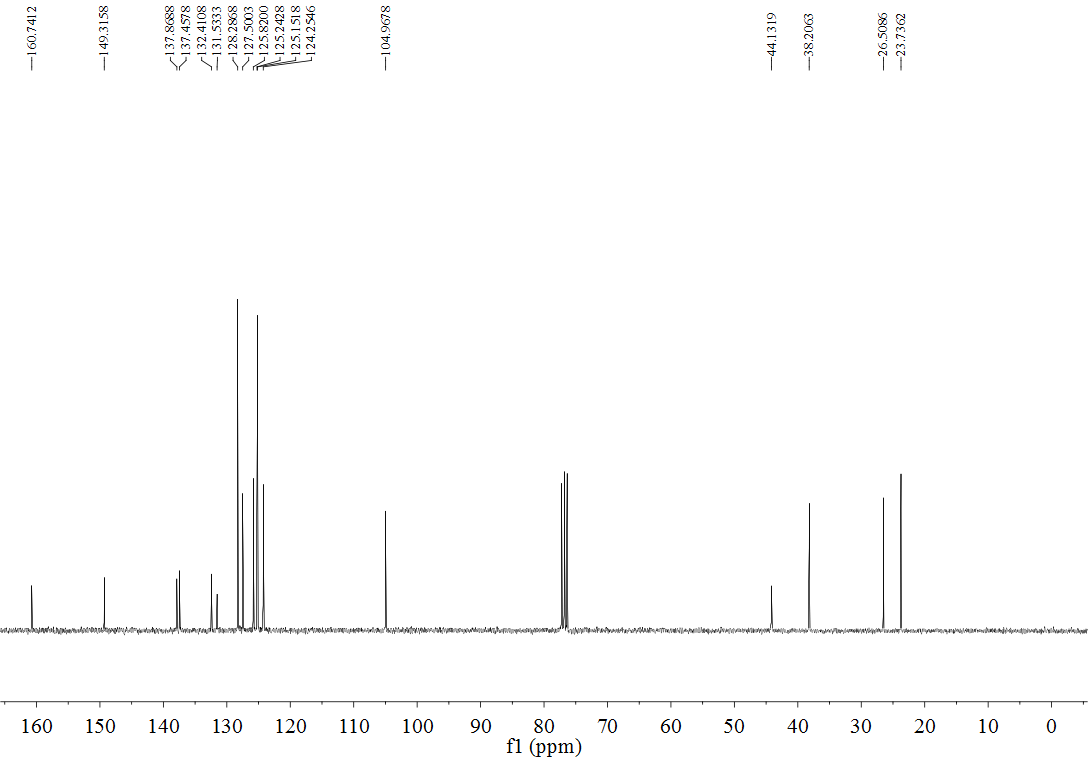

**10e**


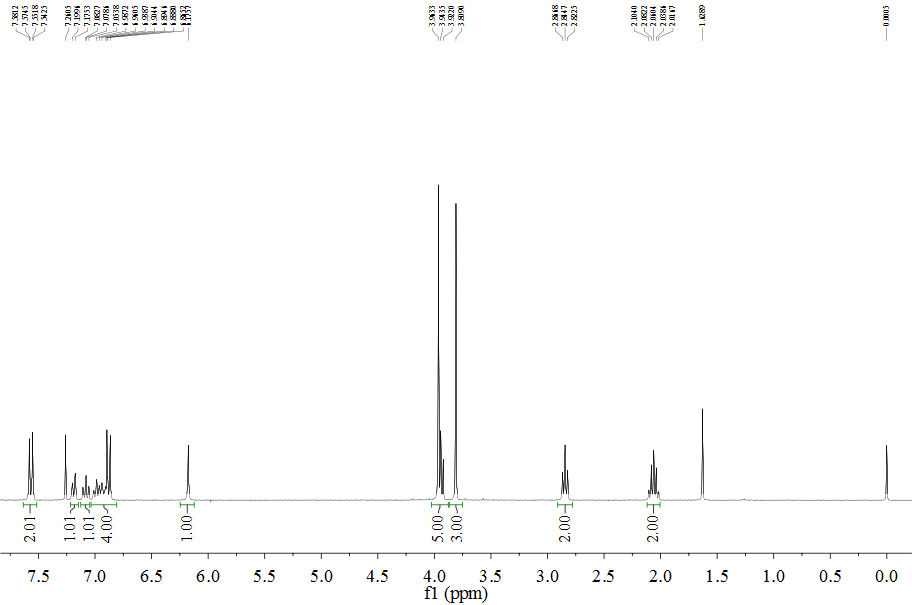

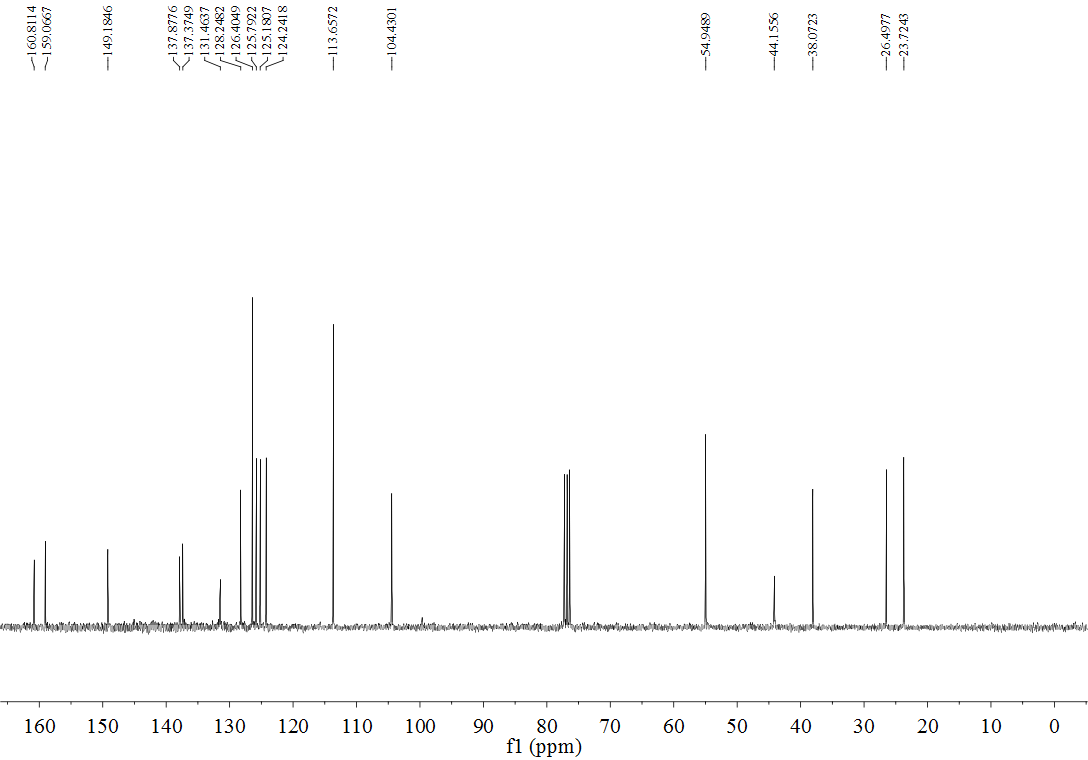

**10f**


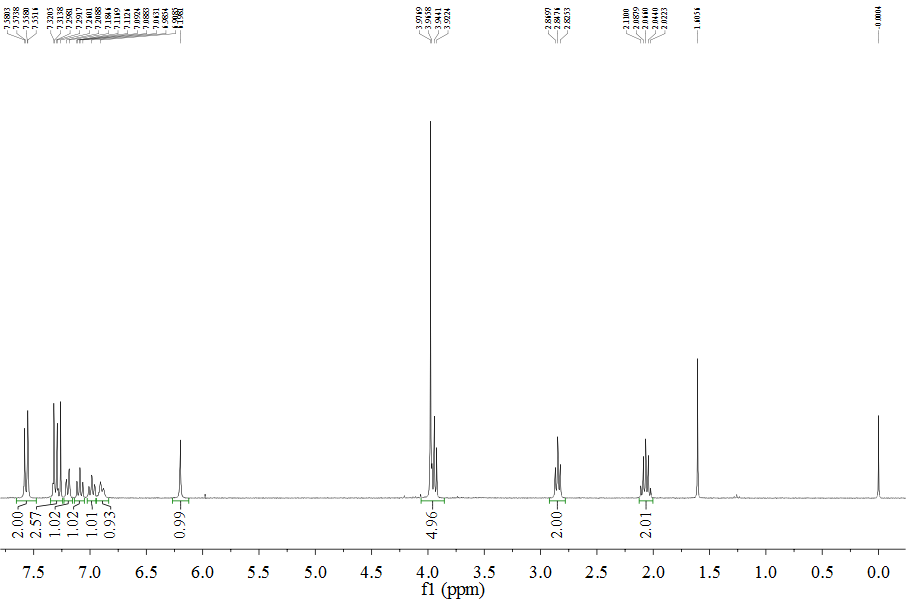

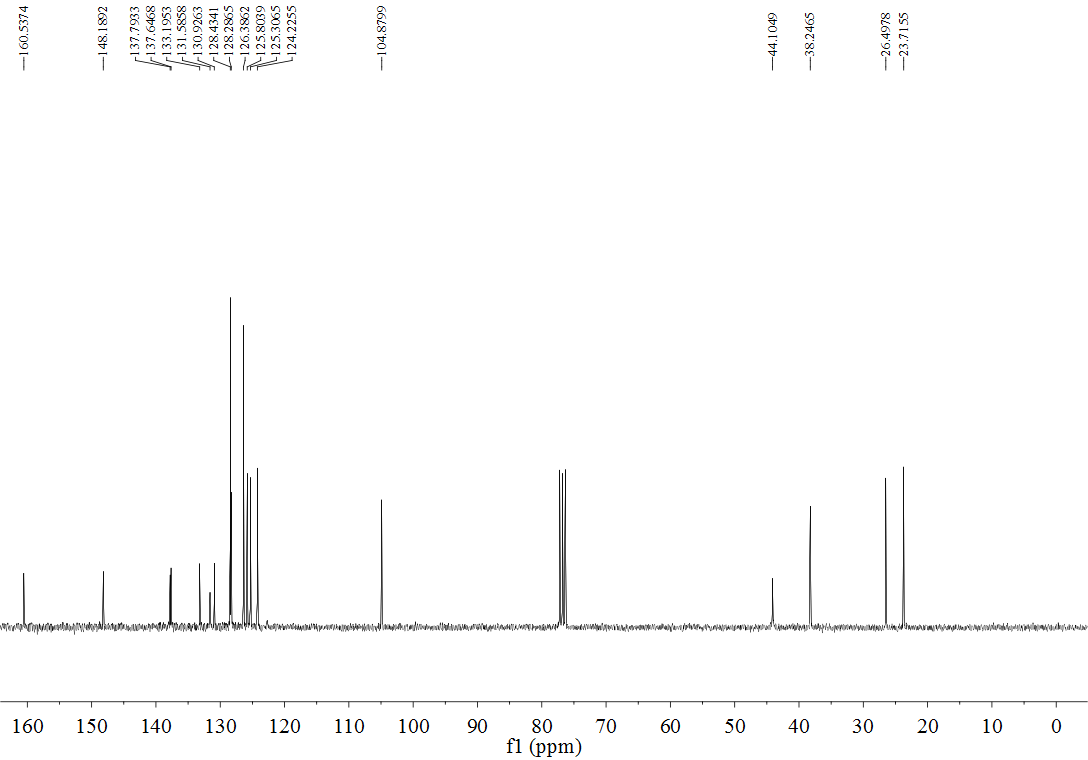

**10g**


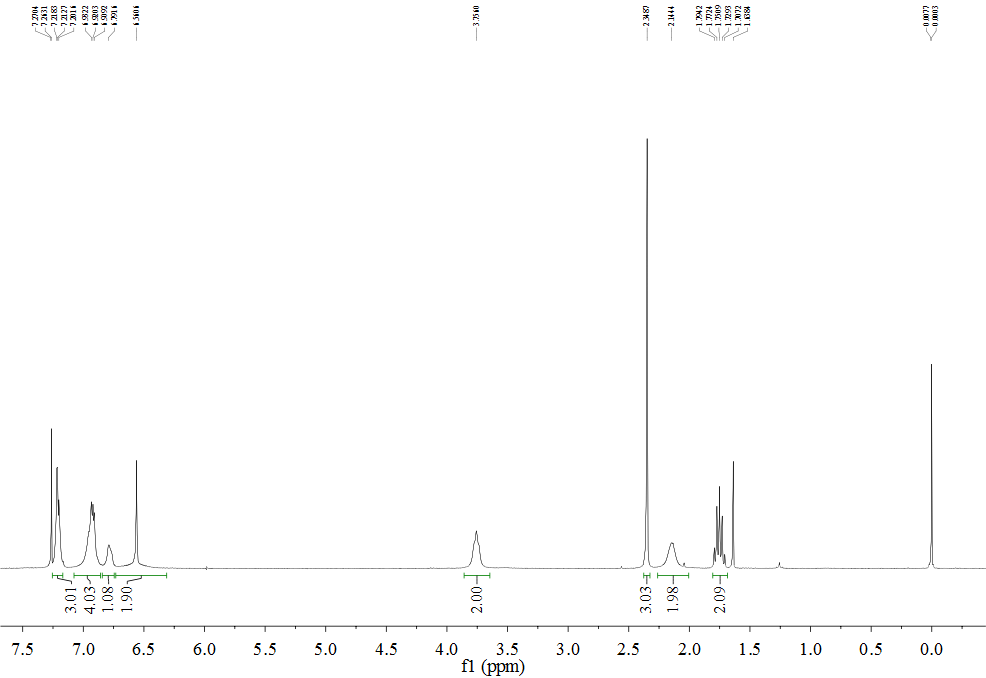

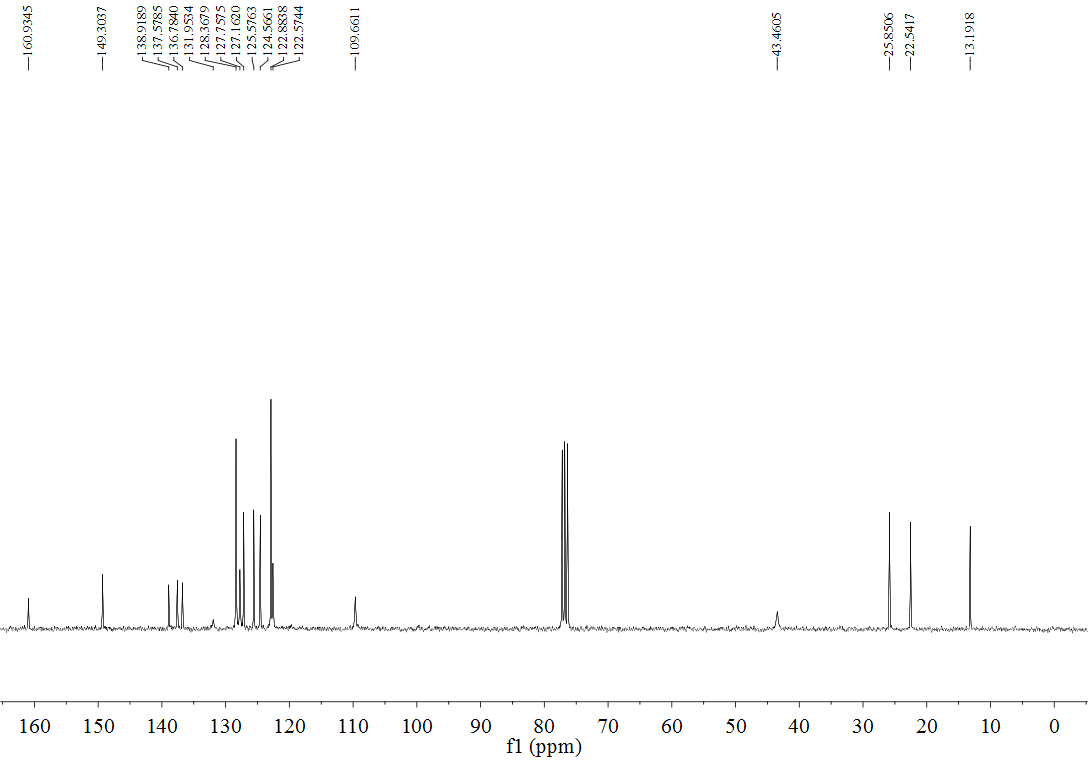

**10h**


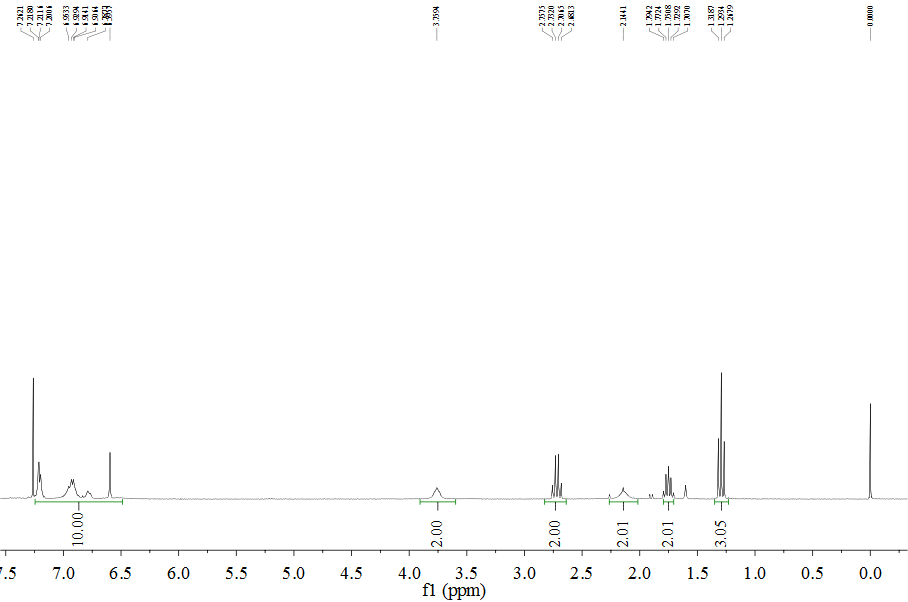

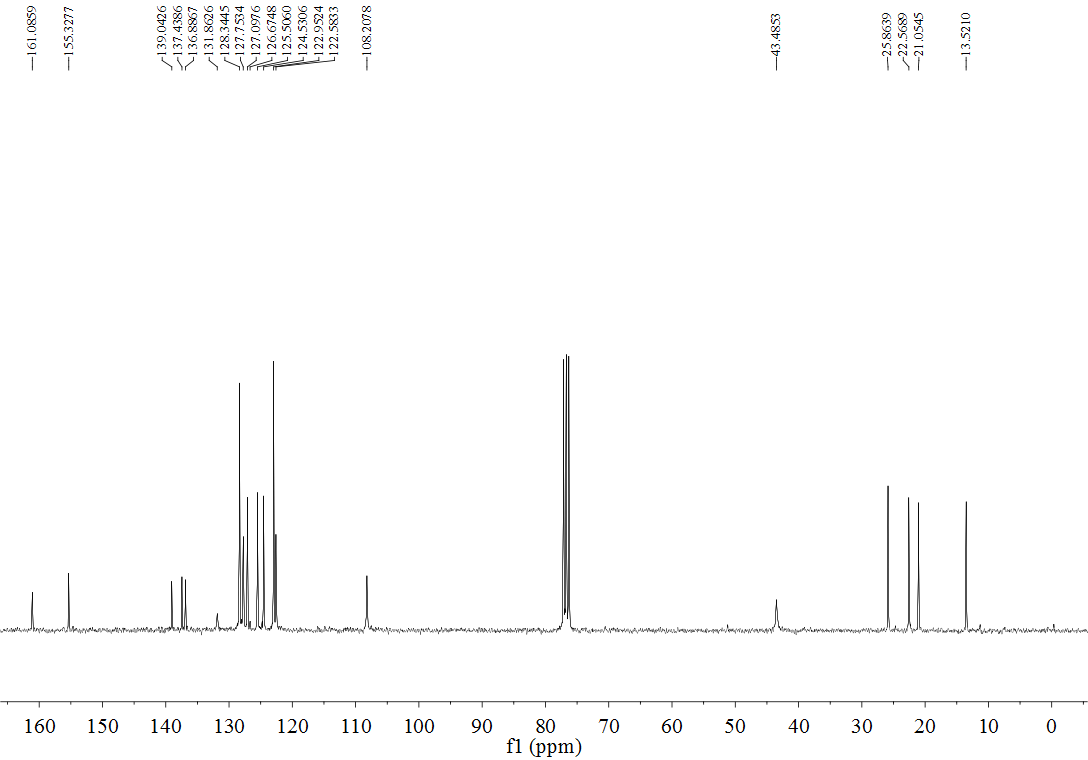

**10i**


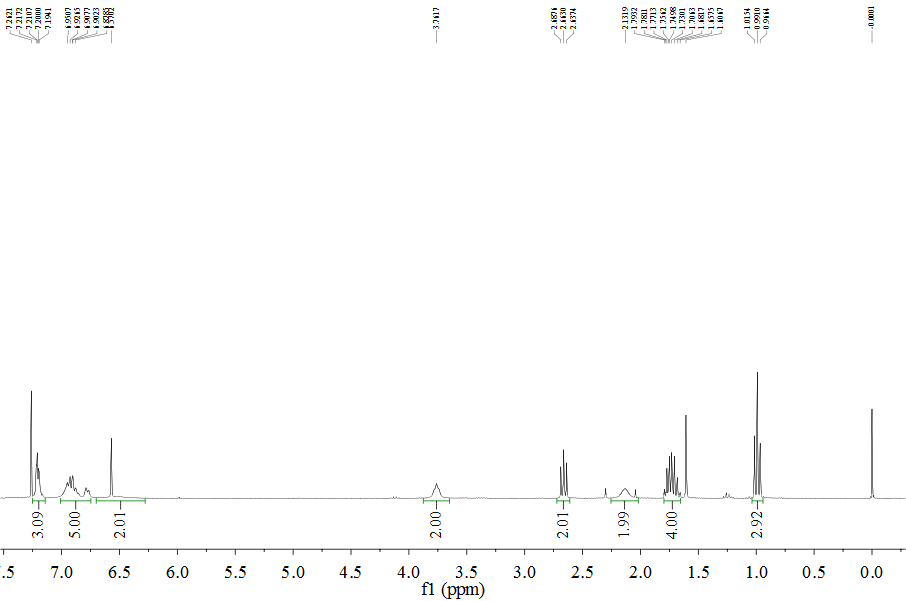

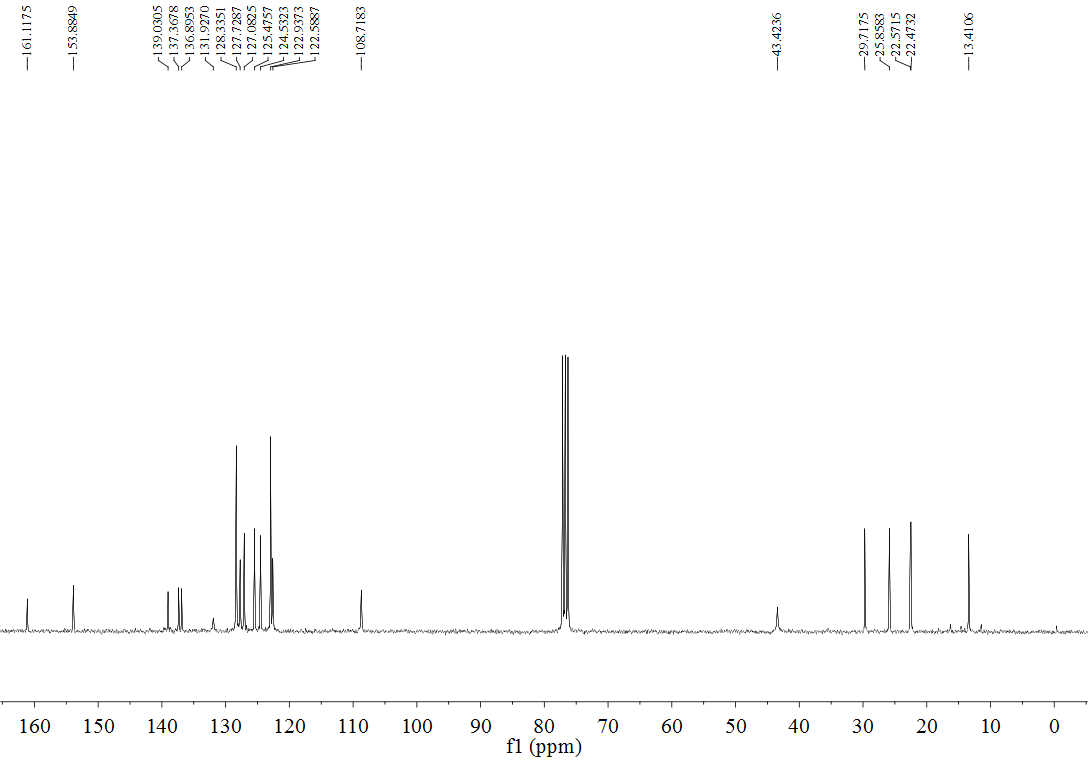

**10j**


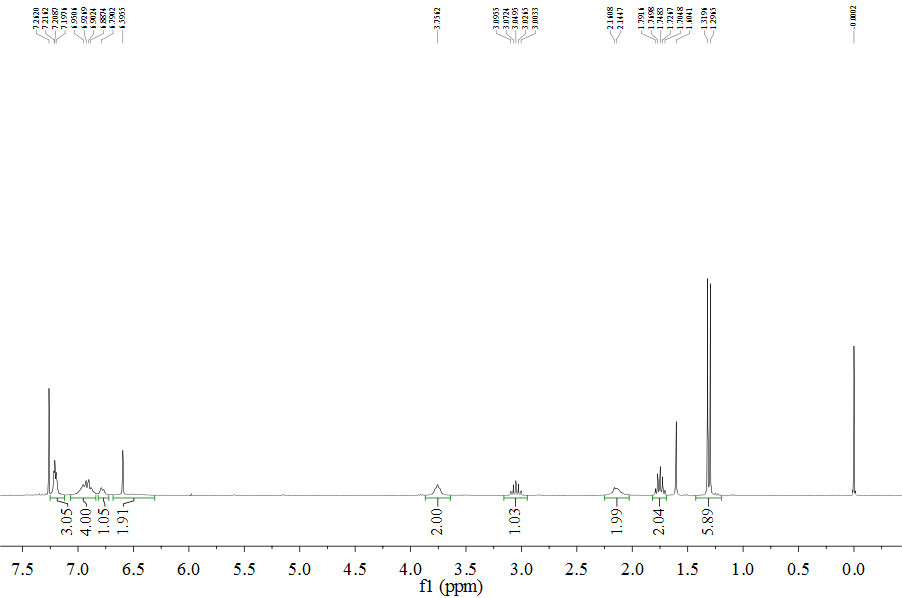

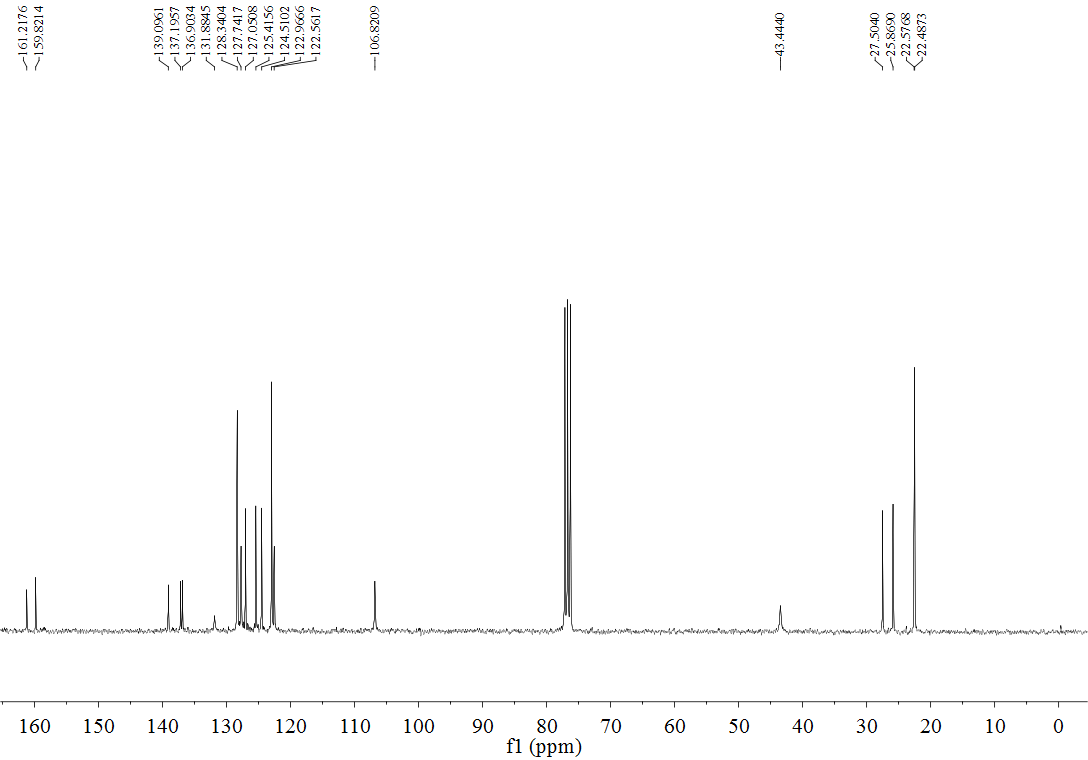

**10k**


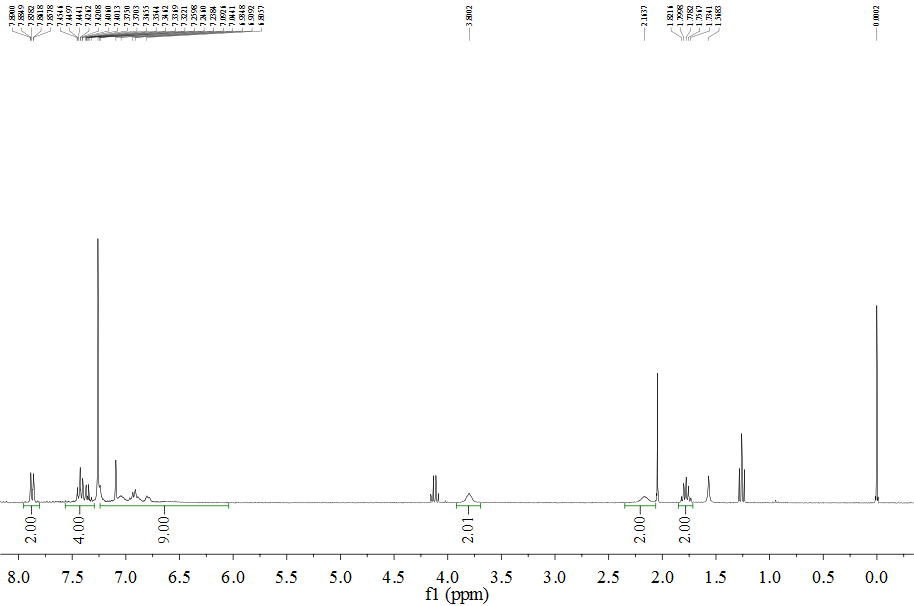

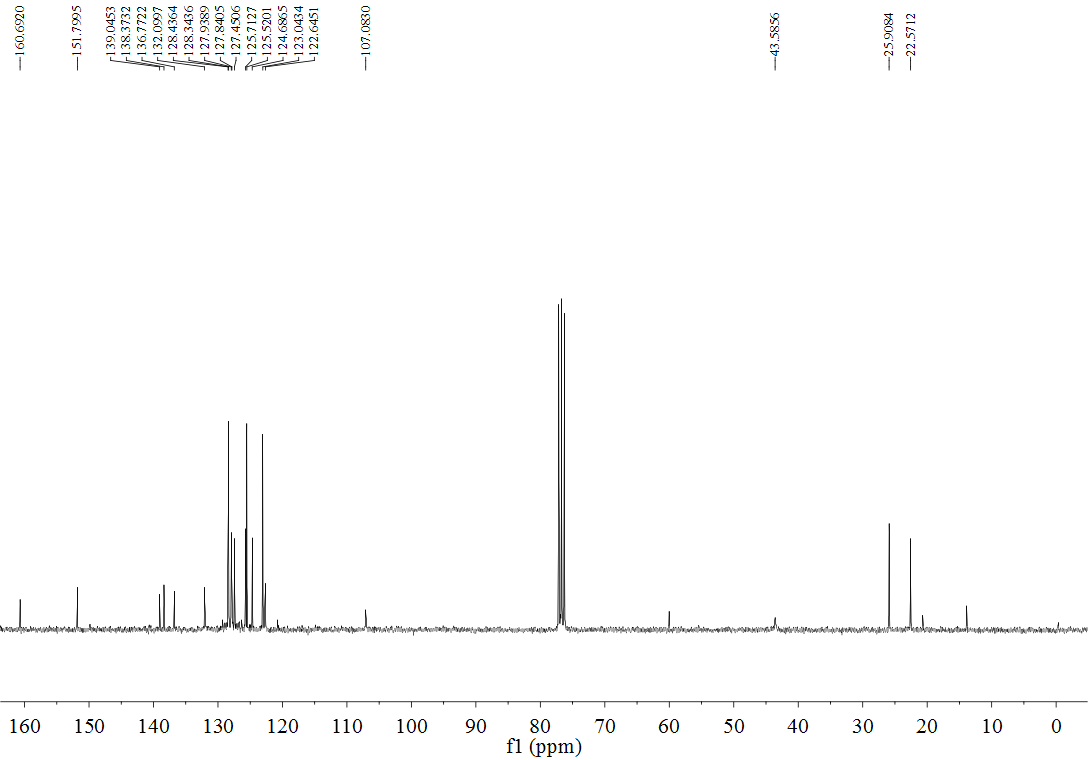

**10l**


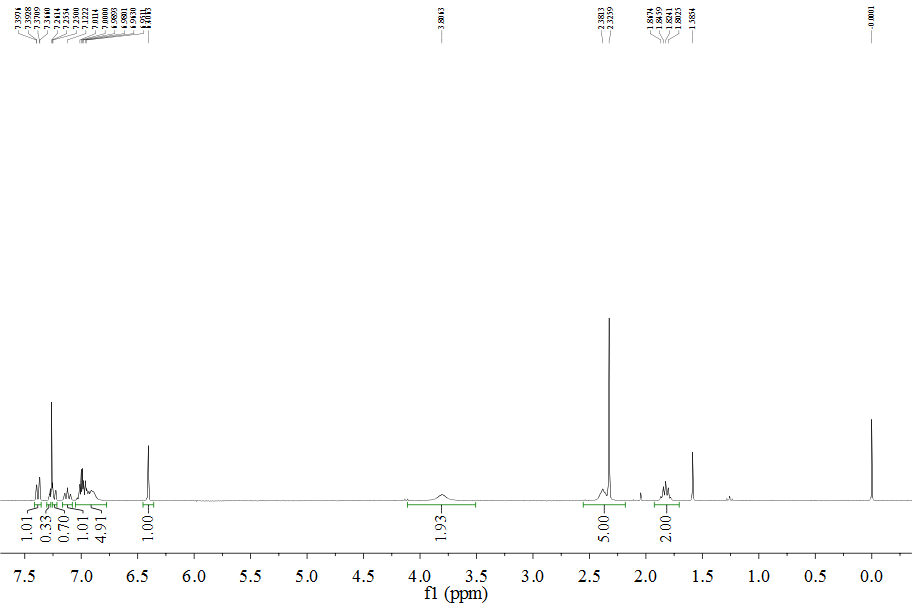

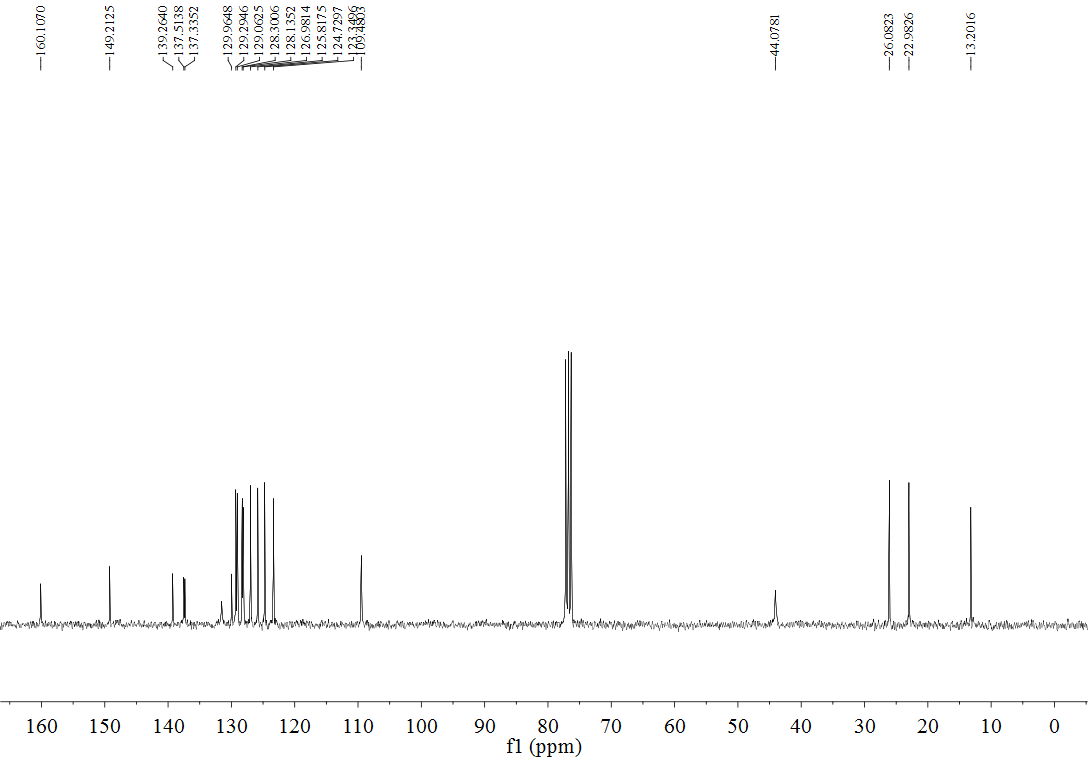

**10m**


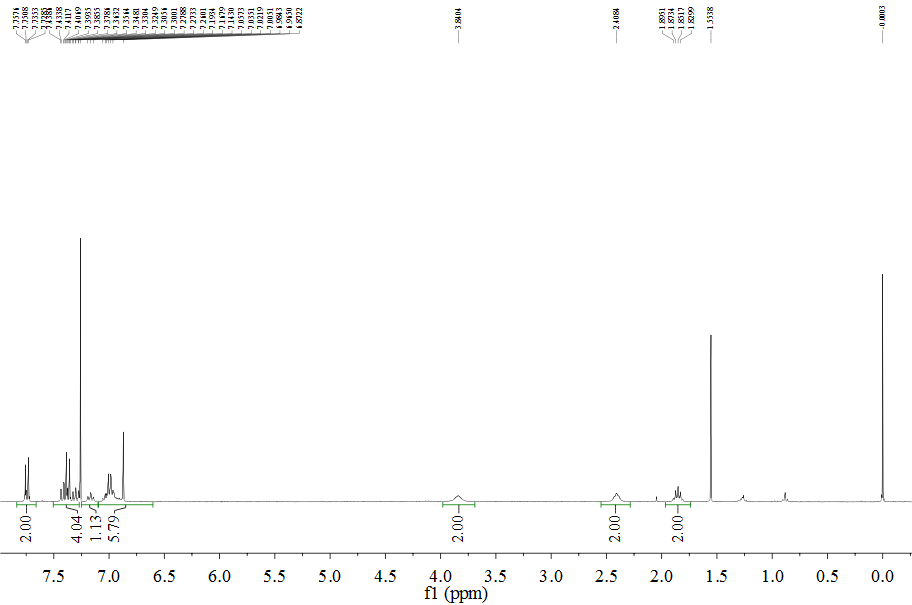

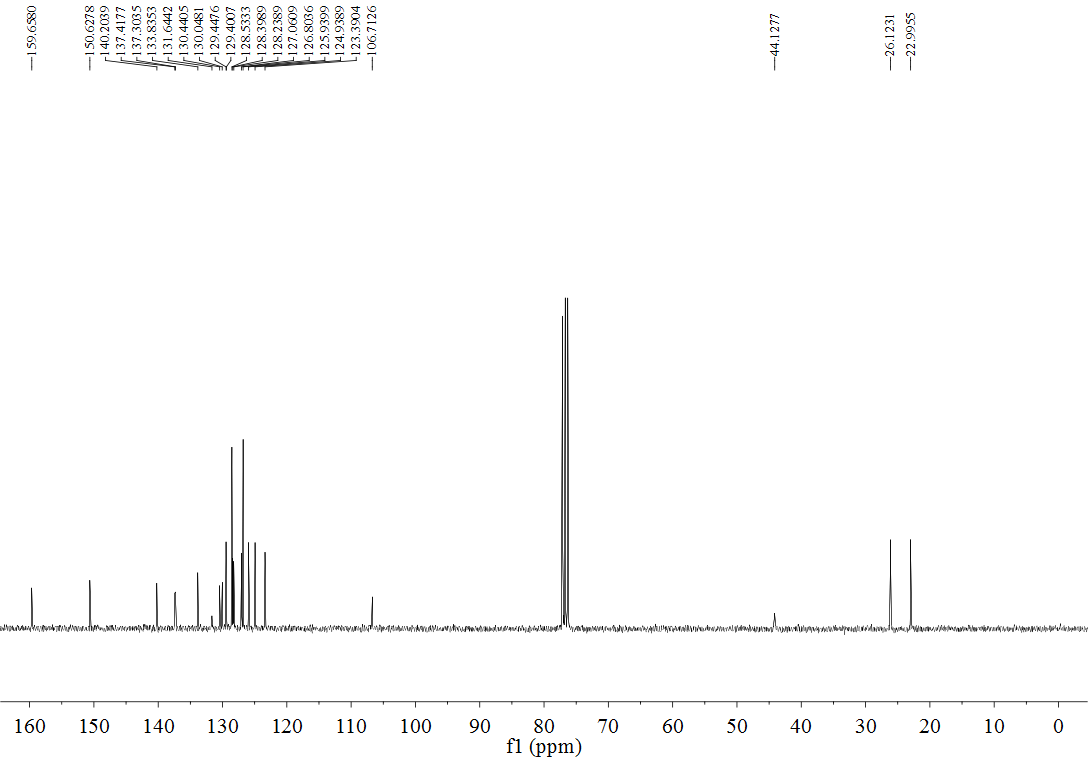

**10n**


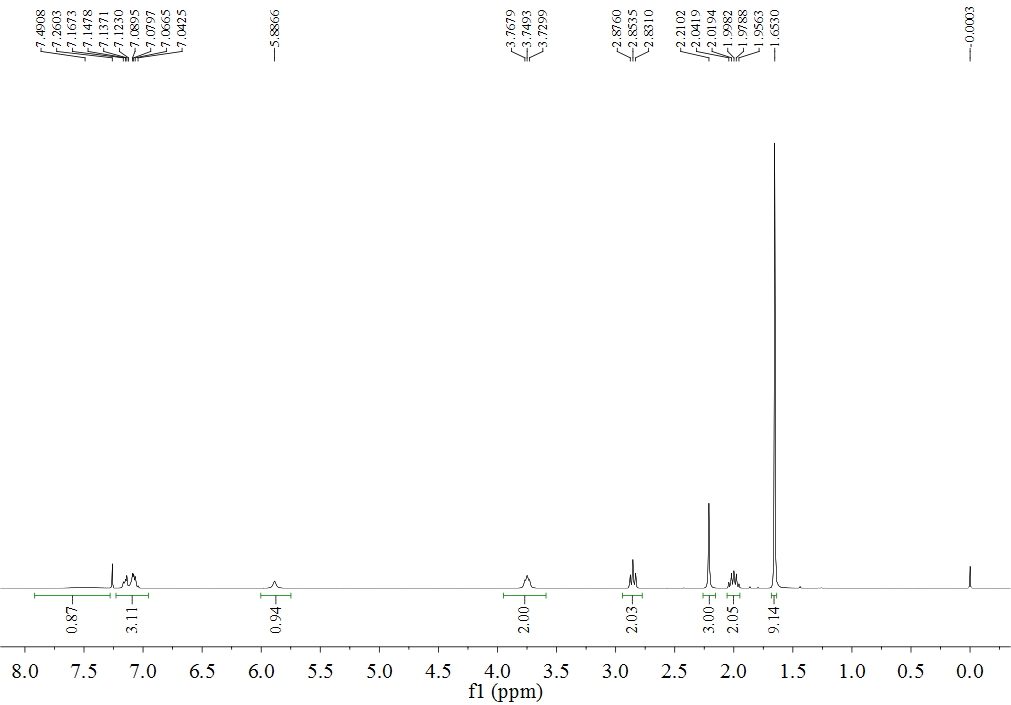

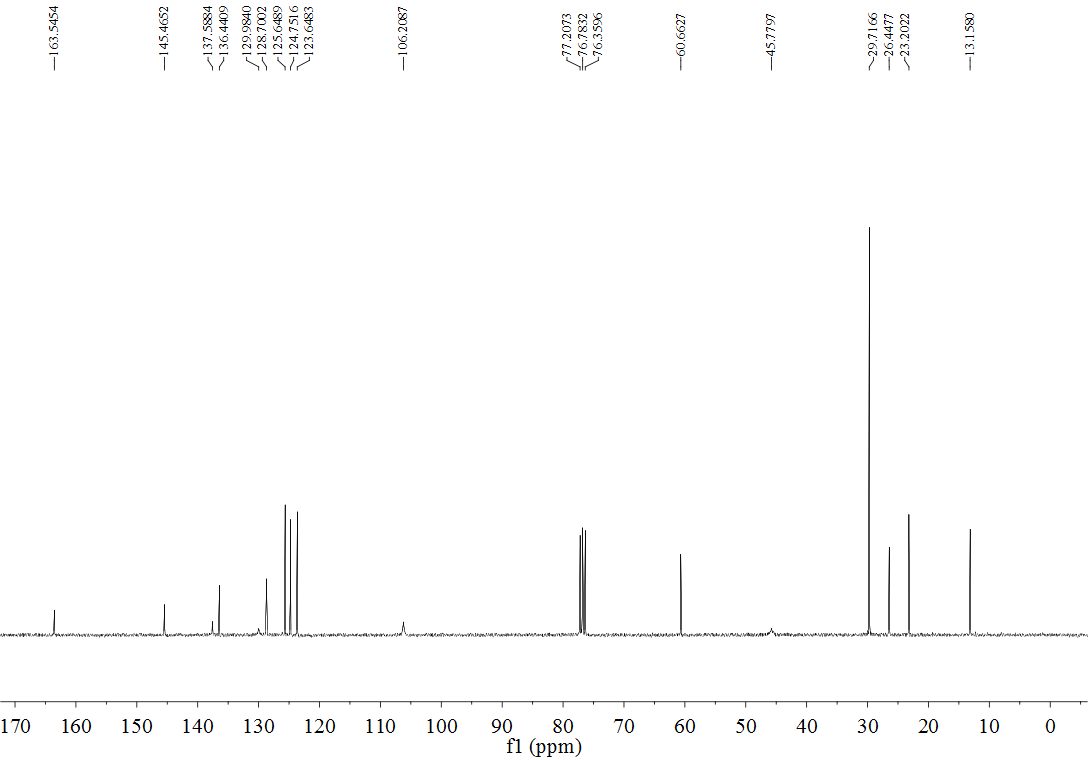

**10o**


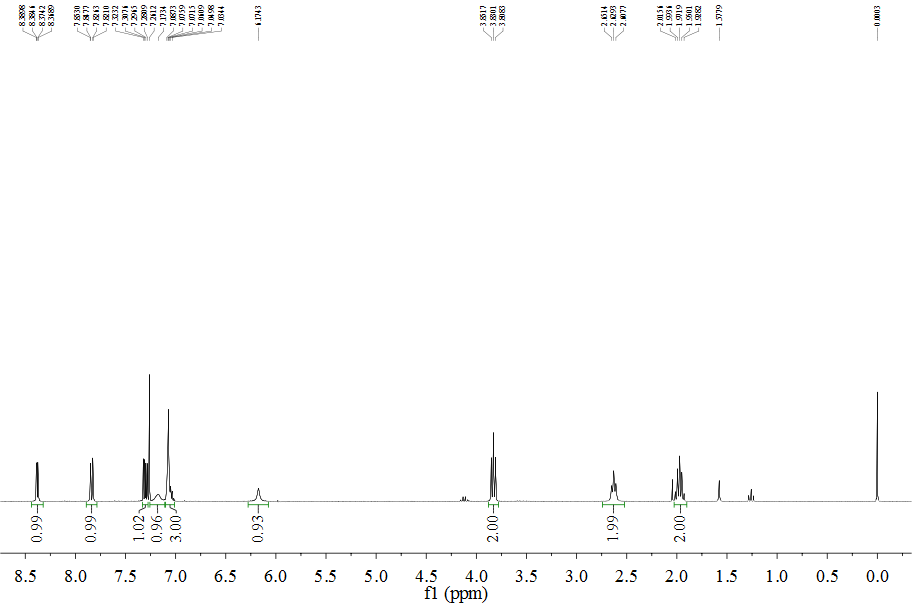

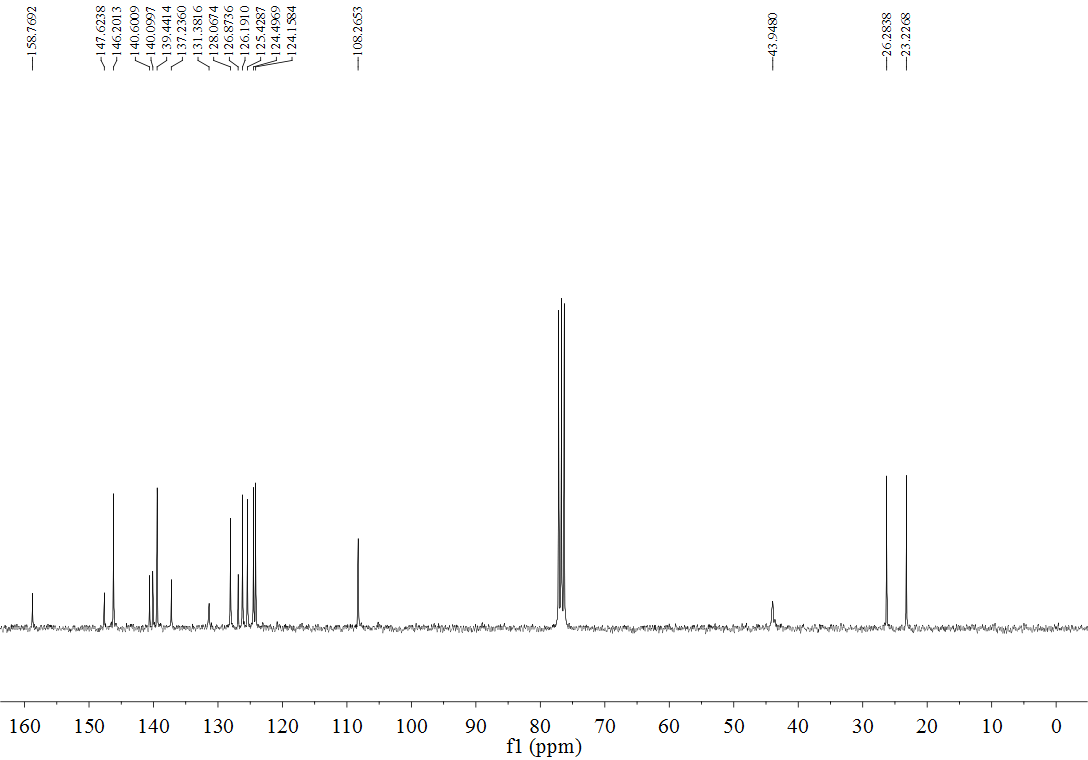

**10p**


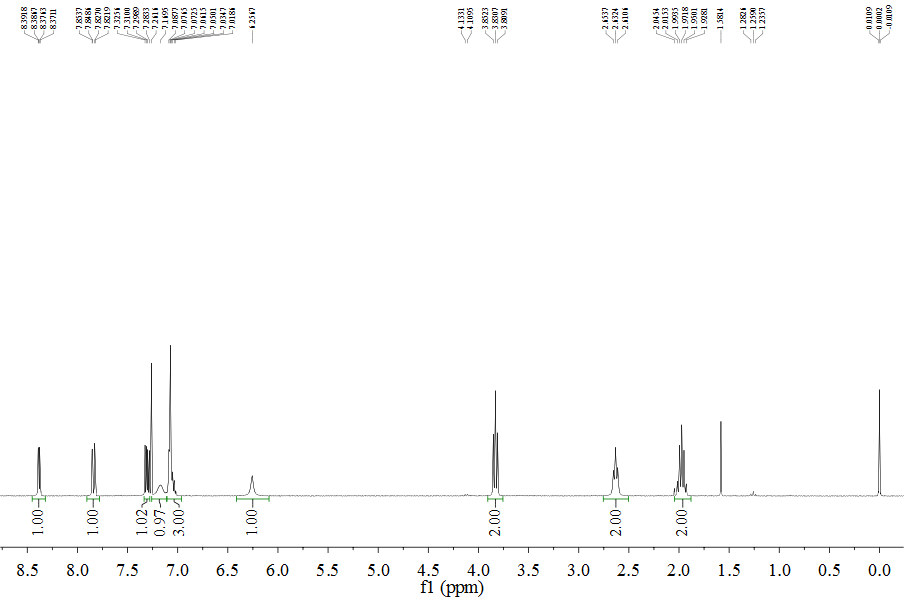

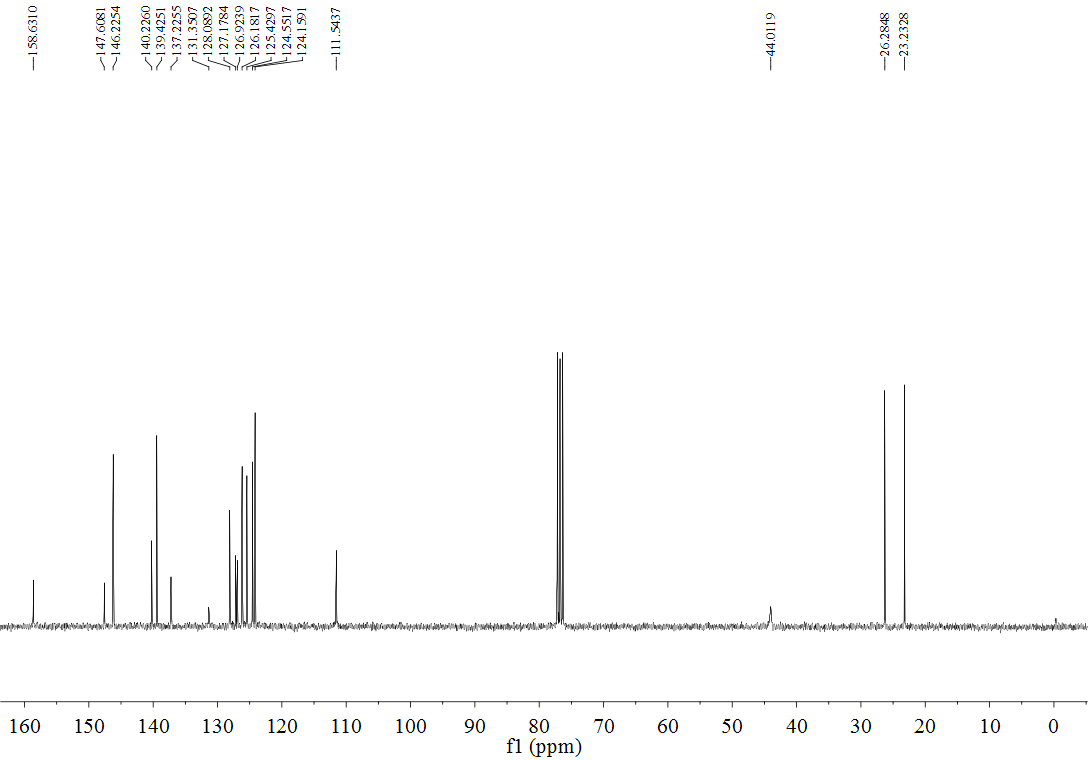

1. * Corresponding: [lyun@cau.edu.cn](mailto:lyun@cau.edu.cn) (Ling Y) [↑](#footnote-ref-1)
