# Supplementary material for: Synthesis and fungicidal activity of pyrazole derivatives containing 1,2,3,4-tetrahydroquinoline
Source: Chem Cent J. 2016 Jul 4;10:40. doi: 10.1186/s13065-016-0186-8 (PMC4932680; doi:10.1186/s13065-016-0186-8)
Supplement: Supplementary file 2 — 10.1186/s13065-016-0186-8 Structure description of the compound 10g. Which includes bond lengths and bond angles. [file 13065_2016_186_MOESM2_ESM.pdf]

Additional file 2

Synthesis and fungicidal activity of pyrazole derivatives containing 1,2,3,4-tetrahydroquinoline

Peng Lei [1], Xuebo Zhang [1], Yan Xu [1], Gaofei Xu [1], Xili Liu [2], Xinling Yang [1], Xiaohe Zhang [1], Yun Ling [1 *]

[1] *Department of Applied Chemistry, College of Science, China Agricultural University, Beijing 100193, China*

[2] *Department of Plant Pathology, China Agricultural University, Beijing 100193, China*

* Corresponding: lyun@cau.edu.cn (Ling Y)

Structure description of the compound 10g

Table S1. Bond Lengths for compound **10g**.

Atom	Atom	Length/Å	Atom	Atom	Length/Å
C1	C2	1.388(3)	C11	C12	1.378(3)
C1	C6	1.397(3)	C11	C16	1.378(3)
C1	N3	1.431(3)	C11	N1	1.425(3)
C2	C3	1.385(3)	C12	C13	1.378(3)
C3	C4	1.381(3)	C13	C14	1.379(4)
C4	C5	1.376(4)	C14	C15	1.380(3)
C5	C6	1.382(3)	C15	C16	1.390(3)
C6	C7	1.500(3)	C17	C18	1.369(3)
C7	C8	1.525(4)	C17	N1	1.367(3)
C8	C9	1.513(3)	C18	C19	1.399(3)
C9	N3	1.479(3)	C19	C20	1.496(3)
C10	C17	1.489(3)	C19	N2	1.329(3)
C10	N3	1.353(3)	N1	N2	1.365(2)
C10	O1	1.229(3)			

Table S2. Bond Angles for compound **10g**.

Atom	Atom	Atom	Angle/°	Atom	Atom	Atom	Angle/°
C2	C1	C6	120.8(2)	C13	C12	C11	119.6(2)
C2	C1	N3	121.84(19)	C12	C13	C14	120.3(2)
C6	C1	N3	117.35(19)	C13	C14	C15	120.0(2)
C3	C2	C1	119.4(2)	C14	C15	C16	120.0(2)
C4	C3	C2	120.0(2)	C11	C16	C15	119.2(2)
C5	C4	C3	120.2(2)	C18	C17	C10	127.5(2)
C4	C5	C6	121.1(2)	N1	C17	C10	125.99(19)
C1	C6	C7	117.9(2)	N1	C17	C18	106.51(19)
C5	C6	C1	118.4(2)	C17	C18	C19	105.7(2)
C5	C6	C7	123.5(2)	C18	C19	C20	128.8(2)
C6	C7	C8	106.97(19)	N2	C19	C18	111.39(19)
C9	C8	C7	110.83(19)	N2	C19	C20	119.7(2)
N3	C9	C8	112.50(19)	C17	N1	C11	129.98(18)
N3	C10	C17	120.4(2)	N2	N1	C11	118.65(17)
O1	C10	C17	118.43(18)	N2	N1	C17	111.37(17)
O1	C10	N3	121.1(2)	C19	N2	N1	104.98(18)
C12	C11	N1	118.8(2)	C1	N3	C9	119.01(16)
C16	C11	C12	120.9(2)	C10	N3	C1	124.88(19)
C16	C11	N1	120.27(19)	C10	N3	C9	116.06(19)
